# Developmental GABA polarity switch and neuronal plasticity in Bioengineered Neuronal Organoids

**DOI:** 10.1038/s41467-020-17521-w

**Published:** 2020-07-29

**Authors:** Maria-Patapia Zafeiriou, Guobin Bao, James Hudson, Rashi Halder, Alica Blenkle, Marie-Kristin Schreiber, Andre Fischer, Detlev Schild, Wolfram-Hubertus Zimmermann

**Affiliations:** 1 Institute of Pharmacology and Toxicology, University Medical Center, Georg-August-University, Göttingen, Germany; 2 DZHK (German Center for Cardiovascular Research), partner site Göttingen, Göttingen, Germany; 3 Cluster of Excellence “Multiscale Bioimaging: from Molecular Machines to Networks of Excitable Cells” (MBExC), University of Göttingen, Göttingen, Germany; 4 Institute of Neurophysiology and Cellular Biophysics, University Medical Center, Georg-August-University, Göttingen, Germany; 5 QIMR Berghofer Medical Research Institute, Brisbane, Australia; 6 Luxembourg Centre for Systems Biomedicine, University of Luxembourg, 4365 Esch-sur-Alzette, Luxembourg; 7 Department for Epigenetics and Systems Medicine in Neurodegenerative Diseases, German Center for Neurodegenerative Diseases (DZNE) Goettingen, 37075 Göttingen, Germany; 8 Department of Psychiatry and Psychotherapy, University Medical Center, Georg-August-University, Göttingen, Germany

**Keywords:** Tissue engineering, Developmental neurogenesis

## Abstract

Brain organoids are promising tools for disease modeling and drug development. For proper neuronal network formation excitatory and inhibitory neurons as well as glia need to co-develop. Here, we report the directed self-organization of human induced pluripotent stem cells in a collagen hydrogel towards a highly interconnected neuronal network at a macroscale tissue format. Bioengineered Neuronal Organoids (BENOs) comprise interconnected excitatory and inhibitory neurons with supportive astrocytes and oligodendrocytes. Giant depolarizing potential (GDP)-like events observed in early BENO cultures mimic early network activity of the fetal brain. The observed GABA polarity switch and reduced GDPs in >40 day BENO indicate progressive neuronal network maturation. BENOs demonstrate expedited complex network burst development after two months and evidence for long-term potentiation. The similarity of structural and functional properties to the fetal brain may allow for the application of BENOs in studies of neuronal plasticity and modeling of disease.

## Abstract (web summary)

Brain organoids are important tools to study early development and disease but little is known of their network activity and plasticity. Here the authors generate iPSC-derived neuronal organoids that display early network formation and maturation with evidence for a GABA polarity switch and long-term potentiation.

## Introduction

Brain organoids recapitulate several aspects of cortical development in vitro. Applications in disease modeling^1,2^ and drug screening^3^ have been demonstrated. Patterning protocols have been developed to generate fore-, mid-, and ventral-brain organoids^4–6^. Although complex anatomical layering and activity of individual neurons has been elegantly demonstrated^7,8^, little is known about network function^9^ and plasticity of established brain organoid models.

During early corticogenesis, bursts of action potentials cause spreading of giant waves of calcium influxes through the developing cortex^10^. This synchronized activity, described as giant depolarizing potentials (GDPs), depends both on excitatory glutamate and GABA inputs^11^. GDPs are initiated by so called hub or pioneer neurons, which are known to have long axons and to connect with many other neurons in parallel^12^. In humans, GABA and glutamate sensitive GDPs have been observed in fetal cortex^13–15^.

A number of studies have shown the importance of supporting glia for neuronal network function and plasticity^16^. Moreover, a recent study on network function of brain organoids elegantly demonstrated the importance of inhibitory neurons for complex network development, both coinciding after 6 months of differentiation^9^.

Aiming to recapitulate human brain network function, we develop Bioengineered Neuronal Organoids (BENOs) from human-induced pluripotent stem cells (iPSCs). BENOs consist of functionally integrated excitatory (glutamatergic) and inhibitory (GABAergic) neurons as well as supporting glia. Collectively, this cellular diversity demonstrates neuronal network function classically found in the developing brain, such as GDP and a GABA polarity switch, and within the more matured human brain, such as neuronal plasticity.

## Results

### BENO generation protocol

**Fig. 1:**
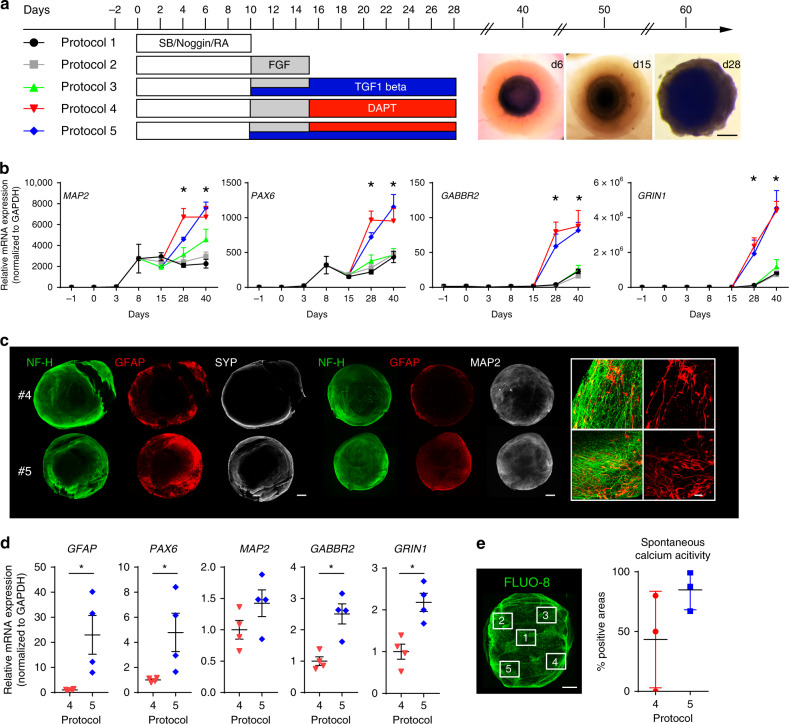
BENO generation from human iPSCs embedded in a defined collagen type I environment. **a** Scheme illustrating the five different protocols tested for their potential to yield the highest amount of neurons and glia in BENO cultures: (1) Dual SMAD inhibition (SB/noggin) in the presence of RA during culture days 0–10; (2) FGF-2 treatment to enhance neuronal progenitor number during culture days 10–15; (3) addition of TGFβ-1 to enhance gliogenesis during culture days 10–28; (4) addition of DAPT to enhance neuronal differentiation during culture days 15–28; (5) combination of protocols 1–4. Right, bright field images of representative BENOs at d6, d15, and d28 of differentiation. Scale bar: 1 mm. **b** Transcriptome analysis of neuronal markers *PAX6, MAP2, GABBR2, GRIN1* during BENO development (d-1, d0, d3, d8), *n* = 3–5 organoids/time point, two independent experiments; all transcripts in protocols 4 and 5 were significantly higher than in protocol 1. *p < 0.0001 two-way ANOVA with Tukey’s multiple comparisons post hoc test. **c** Comparison of protocols 4 and 5 as to their potential to enhance gliogenesis and neuronal maturation. WmIF (whole-mount immune fluorescence) analysis. Scale bar: overview 500 µm, insert 20 µm GFAP was used as a glia marker; **d** transcriptome analysis in d60 BENOs. *n* = 3–5 organoids/time point, two independent experiments, *p* values for *PAX6*, *GABBR2, GRIN1, GFAP* were 0.0485, 0.005, 0.0061, 0.0289, respectively; unpaired two-tailed Student’s *t* test. **e** Comparison of protocols 4 and 5 as to their potential to generate organoids with homogenous distribution of functional neurons. Spontaneous calcium imaging after loading with Fluo-8-AM in different areas of BENOs was performed at d30 by confocal microscopy *n* = 3 organoids/group; Scale bar 500 µm. Data are presented as mean values ±  SEM.

BENOs are generated from human iPSCs dispersed in a collagen type I environment and are subjected to a staged directed differentiation and maturation protocol. The introduced BENO protocol differs from previously established neuronal organoid cultures^1–3,5,6,8^ by (1) a 1-step culture, directed differentiation approach and (2) the use of fully defined components, such as a pluripotent stem cell source, collagen, small molecules, and growth factors. We initially tested five different directed differentiation protocols (Fig. 1a, Supplementary Fig. 1a–c), starting with a 10 day induction of neuroectodermal commitment in the presence of SB/noggin (dual smad inhibition) and retinoic acid (RA) followed by extended culture in Neurobasal medium (protocol 1); further addition of FGF-2 on culture days 10–15 to enhance neuronal progenitor growth^17^ (protocol 2), TGFβ1 on culture days 10–28 to enhance gliogenesis^18^ (protocol 3), and DAPT from culture days 15–28 to enhance neuronal differentiation^19^ (protocol 4) were tested. Finally, a combination of SB/Noggin/RA (days 0–10), FGF-2 (days 10-15), TGFβ1 (days 10-28), and DAPT (days 15-28) was investigated (protocol 5) and found to optimally induce neuro- and gliogenesis. qPCR analyses confirmed a rapid loss of pluripotency (*POU5F1*; Supplementary Fig. 2) with transcripts related to neurogenesis (*PAX6, MAP2, GRIN1, GABBR2*) being upregulated from culture day 15 onwards in protocols 4 and 5 (Fig. 1b, Supplementary Fig. 2). This was confirmed by immunostaining for neuronal markers (*NF-H, SYP, MAP2*) (Fig. 1c). While both protocols supported neurogenesis, protocol 5 additionally enhanced gliogenesis (Fig. 1b–d, Supplementary Fig. 1d) and increased reproducibility as indicated by robust spontaneous calcium activity (Fig. 1e) in morphologically highly uniform BENOs at all investigated time points (Supplementary Fig. 1e).

### Transcriptome profiling during BENO development

**Fig. 2:**
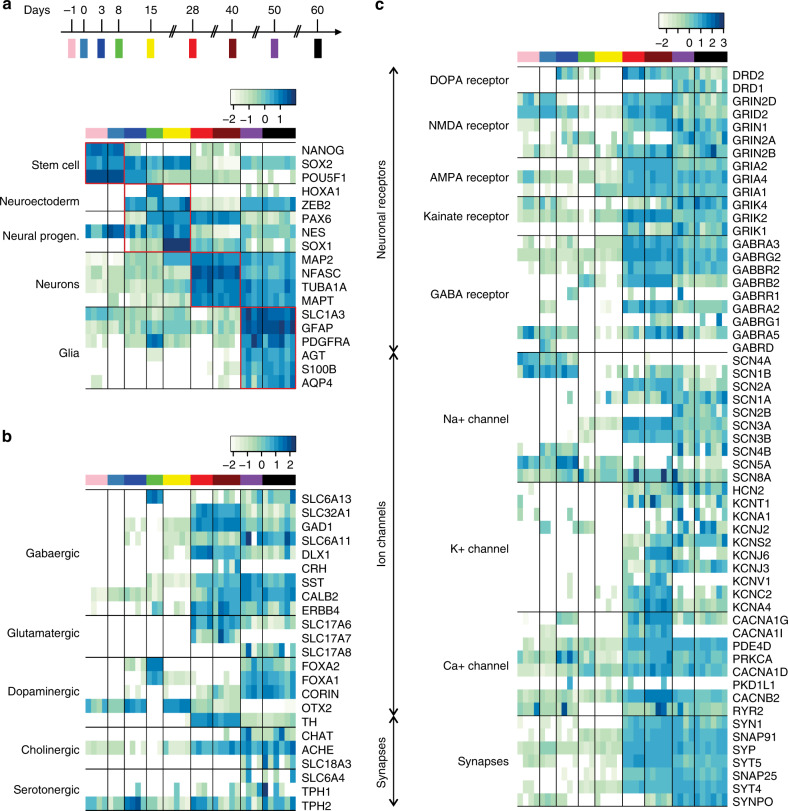
Transcriptome analyses during BENO generation (from d-1 to d60) by RNA sequencing. **a** Heat map showing distinct developmental stages: d-1 to d0—pluripotent stem cell stage; d3–d8—neuroectodermal commitment stage; d8–d15—NPC stage; d15–d40—neurogenesis; d40 and onwards—neuronal maturation with concurrent gliogenesis. **b** Increase of the abundance of transcripts encoding for GABAergic, glutamatergic, dopaminergic, cholinergic, and serotonergic proteins from d28 onwards. **c** Heat map depicting the expression of transcripts encoding for proteins involved in synaptic transmission, including postsynaptic receptors, ion channels, and synaptic proteins.

To gain insight into the cell population dynamics during BENO formation, we performed a time-course RNAseq analysis from samples obtained on culture days 0, 3, 8, 15, 28, 40, 50 and 60 (two independent experiments, *n* = 3–6 organoid/time point). Principal component analyses and correlation heat maps provided evidence for the recapitulation of distinct developmental stages in BENOs on culture days d-1 to d3, d3–d8, d8–d15, d28–d40, and d50–d60 (Supplementary Fig. 3a, b). Notably, BENOs presented a low inter-organoid variability at the different sampling time points. A prominent expression of pluripotency markers characterized the cell population between d-1 and 3. On d3–8, BENOs were enriched in early neuroectodermal cell markers, on d8–15 in neuronal progenitor cell (NPC) markers, on d28–40 in neuronal markers, and on d50–60 in glia markers (Fig. 2a). Between d28–60, markers expressed by distinct types of neurons (glutamatergic, GABAergic, catecholaminergic), their respective receptors as well as transcripts related to synaptic transmission and ion channels were abundantly identified (Fig. 2b, c). In line with this observation, gene ontology (GO) analyses in d15 vs. d40 BENOs indicated the presence of components, which are essential for neuronal network function and plasticity (Supplementary Data 1). Endodermal and mesodermal markers showed minimal expression (5–10 FPKM) compared to neuronal markers (100–1000 FPKM; Supplementary Fig. 3c) detailing that BENOs contained negligible non-ectodermal components. From d50, transcripts indicating the development of outer radial glia were strongly upregulated along with hyaluronic acid synthase, hyaluronan, proteoglycan link protein 1, lumicon, and collagen I, a subset of proteins which recently have been shown to be involved in the folding of embryonic human cortex^20^ (Supplementary Fig. 3d). At the same time, the high expression of astrocyte markers was accompanied by extracellular matrix components, suggesting the presence of functional astrocytes (Supplementary Fig. 3e).

### BENO cellular composition

**Fig. 3:**
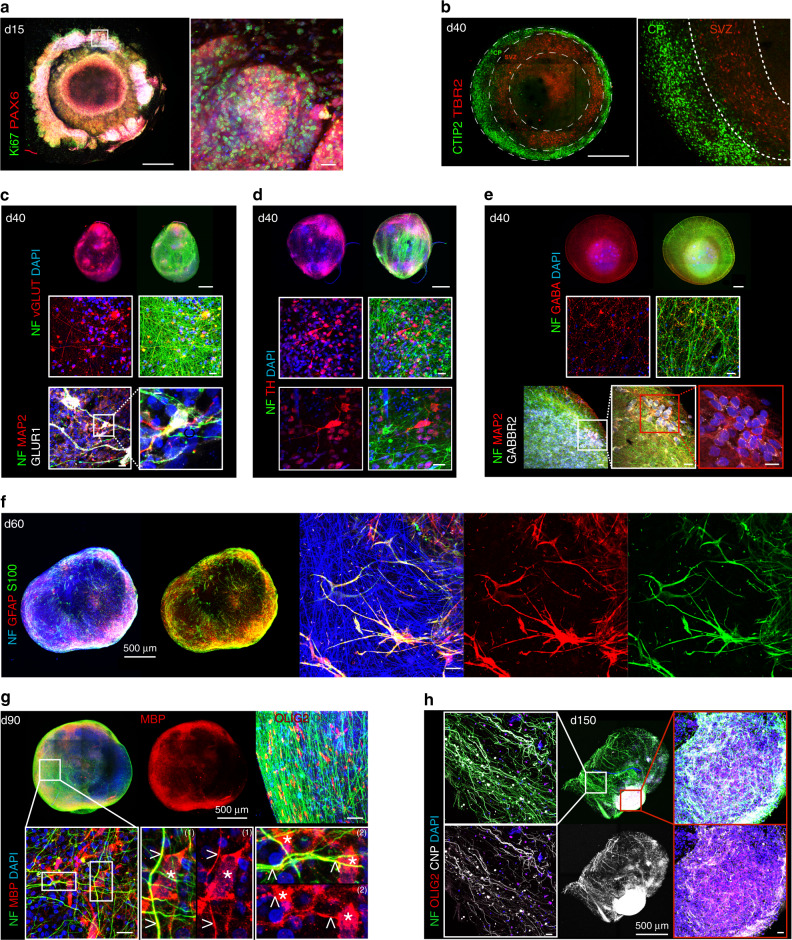
Cellular composition of BENOs in different times of differentiation. **a** Representative overview and higher magnification images of indicated region of a d15 BENO showing proliferating NPCs (PAX6^+^/Ki67^+^; refer to Supplementary Fig. 4a for the individual confocal planes). **b** Distinguishable concentric surrogate subventricular zone (TBR2^+^) and a cortical plate (CTIP2^+^) in d40 BENO. **c**–**e** Excitatory (vGLUT^+^, TH^+^) and inhibitory (GABA^+^) neurons as well as their respective receptors (GLUR1, GABBR2) in d40 BENOs. **f** Overview (left) of a d60 BENO stained for astrocyte markers (GFAP^+^/S100β^+^) and neuronal axons (NF^+^); close-up views (right) of GFAP^+^/S100β^+^ cells with typical astrocyte morphology. **g** Detection of oligodendrocyte progenitors (OLIG2^+^) and oligodendrocytes (MBP^+^/CNP^+^) in d90 BENO. Inserts 1 and 2 highlight MBP^+^ oligodendrocytes (*), which contribute to myelination of axons (<). **h**, On d150 a higher number of mature oligodendrocytes and myelinated axons appeared. All data presented on this figure derive from at least three independent experiments with similar results. Scale bars: overviews 200 µm, close-up views 20 µm, unless indicated otherwise.

Next, we utilized whole-mount immunofluorescence (WmIF) analyses to investigate the spatiotemporal development of multiple cell populations in BENOs. On d15, we observed concentric expansion of highly proliferative PAX6^+^ Ki67^+^ NPCs (Fig. 3a). On d40, evidence for the development of a subventricular zone (TBR2^+^ cells) adjacent to an outer self-organizing cortical plate (CTIP2^+^ cells) was obtained (Fig. 3b). On d40, BENOs contained excitatory catecholaminergic (TH^+^) and glutamatergic (vGLUT^+^) neurons as well as the respective target cells expressing typical cortical glutamatergic receptors such as GLUR1 (Fig. 3c, d, Supplementary Fig. 4). In parallel, an extensive network of GABA^+^ cells as well as GABBR2^+^ cells were present in d40 BENOs (Fig. 3e). On d60, in line with the transcriptome data, BENOs were enriched with astrocytes marked by GFAP/S100beta expression (Fig. 3f), while first oligodendrocyte progenitors (OLIG2^+^ cells) appeared (Supplementary Fig. 5). By a further extension of BENO culture for up to 150 days, we observed OLIG2^+^ cells as well as MBP and CNP expression and the presence of the first myelinated axons after 90 days in culture (Fig. 3g). By day d150 the number of CNP^+^ OLIG2^+^ cells as well as myelinated axons increased markedly (Fig. 3h, Supplementary Fig. 5b–f).

### GABA- and glutamate-dependent GDP-like events in BENO

We next confirmed the presence of functional neurons by whole-cell patch-clamp recordings of BENO (d35-d65) cultures, showing tetradotoxin (TTX)-sensitive excitatory postsynaptic potentials (TTX, 1 nM) and functional sodium and potassium channels (Supplementary Fig. 6a). To further characterize neuronal network development, we analyzed spontaneous and stimulation-induced calcium activity in BENOs between culture day 20 and 98. Interestingly, the BENO network analysis showed pronounced differences during BENO development, indicating three distinct stages of network development: (1) early stage (ES) prior to d25, (2) intermediate stage (IS) between d25–d40, and (3) late stage (LS) d40 and onwards.

**Fig. 4:**
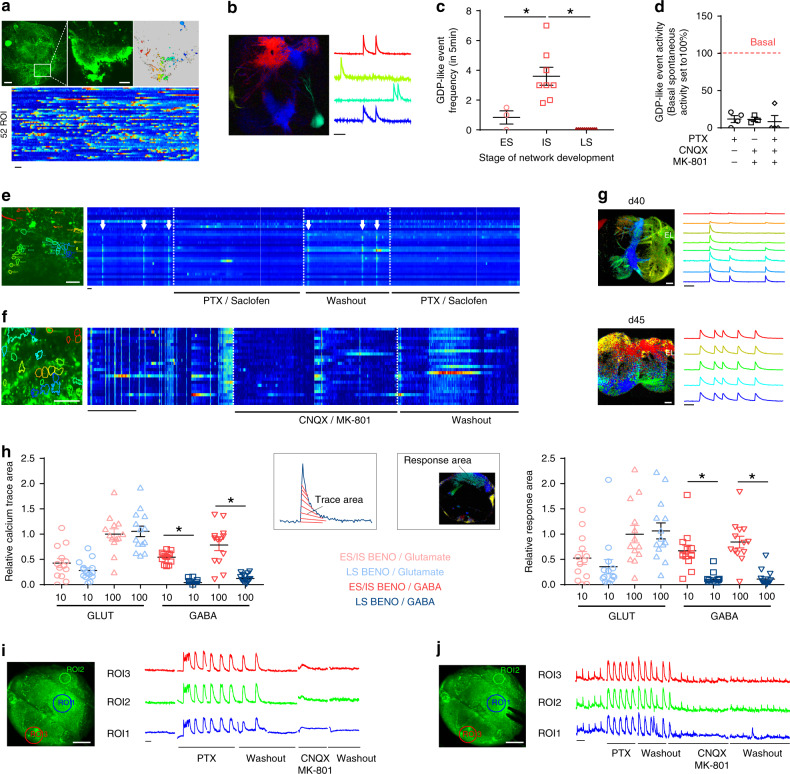
Development of neuronal networks during BENO generation. **a** Calcium activity in d21 BENO. Upper-left: overview of the complete organoid loaded with the calcium indicator Fluo-8-AM. Upper-middle: close-up view of the boxed area. Upper-right: active correlation map of different regions of interest (ROI) showing calcium activity; each ROI is representative for a specific neuronal activity. Bottom: heat map of calcium activity of 52 neurons as a function of time. **b** Active correlation map of d35 BENO and color-coded calcium traces (Δ*F*/*F*0) showing GDP-like events (refer also to Supplementary Videos). **c** GDP-like event frequency at early stage (ES, *d* < 25), intermediate stage (IS, 25 < *d* < 40), late stage (LS, 40 < *d* < 65); *n* = 15 organoids, 2 independent experiments, * *P* values for ES vs IS and IS vs LS were 0.0035 and <0.0001, respectively, one-way ANOVA with Sidak’s multiple comparisons post hoc test. **d** GDP-like event inhibition by PTX, CNQX, and MK-801; *n* = 3-4 organoids/group, 2 independent experiments. **e**, **f**, close-up view showing synchronized calcium activity (indicated by arrows) of individual neurons in d36 BENOs. Heat maps of 20–30 ROIs showing desynchronization upon GABA receptor blockade by PTX/Saclofen (**e**) and glutamate receptor blockade by CNQX/MK-801 (**f**); note that upon washout synchronicity was restored. **g** Overview of calcium activity propagation induced by single pulses (100 µA) in the same fused BENOs on d40 (1 mm signal propagation relative to stimulation electrode) and on d45 (2 mm signal propagation relative to stimulation electrode). **h** In ES and IS BENOs, GDP-like events were evoked by 10 and 100 µmol/L GABA or 10 and 100 µmol/L glutamic acid (calcium signals recorded from 3 independent organoids; symbols indicate data from 15 ROIs). In LS BENOs GDP-like events were evoked by glutamate, but not GABA (calcium signals recorded from 3 organoids of 1 experiment; symbols indicate data from 15 ROIs). Bar graphs summarize calcium trace area (left) and signal strength in response area (right), indicating the extent of the neuronal activity in the investigated BENO. Values were normalized to 100 µmol/L glutamate responses in ES/IS and LS organoids; For relative trace area the *p* values of 10 and 100 µmol/L GABA ES vs LS were 0.002 and <0.0001, respectively. For relative response area the *p* values of 10 and 100 µmol/L GABA ES vs LS were 0.027 and 0.0011, respectively; one-way ANOVA with Tukey’s multiple comparisons post hoc test. **i** Representative traces of spontaneous calcium activity under GABAergic and glutamatergic inhibition in LS BENOs (representative image from ROIs recorded in 3 BENOs). **j** Electrical stimulation-induced calcium activity under GABAergic and glutamatergic inhibition in LS BENOs (representative image from ROIs recorded in 1 of 3 BENOs, *n* = 3 organoids, 2 independent experiments). Spontaneous and induced activity was enhanced under GABA receptor blockade, while induced activity under glutamatergic inhibition was reduced. Upon washout the network balance was restored. Scale bars: Overview 200 µm; high magnification 50 µm. All time scale bars on calcium traces are 30 s. Data are presented as mean values ± SEM.

During the ES, calcium signal analysis demonstrated a high abundance of neurons with TTX-sensitive spontaneous activity (Fig. 4a, Supplementary Fig. 6b). In the next couple of days, the appearance of highly organized, spontaneous, synchronous calcium bursts, resembling GDPs, suggested early network formation similar to processes identified in the developing brain (Fig. 4b, Supplementary Movie 1). At this stage, local electrical stimulation (single pulses, 300 µA) induced TTX-sensitive synchronized calcium bursts at up to 200 µm distance from the electrode, but failed to propagate to remote regions. In contrast, high frequency stimulations (HFS) in ES BENOs evoked GDP-like events throughout the organoid (Supplementary Fig. 6c).

During the IS, spontaneous GDP-like event frequency was the highest (3.6 ± 0.6 events per 5 min, *n* = 8; Fig. 4c, Supplementary Movies 2 and 3). To understand which neurotransmitter contributed to network synchronization, we measured the spontaneous activity of BENOs in the presence and absence of selective inhibitors against GABAergic and glutamatergic receptors. GABAergic inhibition, by the non-selective antagonist for GABA-A/-C, Picrotoxin (PTX: 58 µM) and the selective antagonists for GABA-B, Saclofen (330 µM) reduced the occurrence of GDP-like events (*n* = 4 organoids; Fig. 4d, e, Supplementary Fig. 6d). The reduced activity suggested an existence of excitatory presynaptic GABA signals, which have been recognized to trigger GDPs in the developing brain^12^. Inhibition of the glutamatergic network, by the combination of the non-competitive NMDA antagonist, Dizocilpine (MK-801: 0.2 µM) and the competitive AMPA/kainate receptor antagonist, Cyanquixaline (CNQX: 15 µM), strongly decreased the frequency of synchronous calcium activity (*n* = 3 organoids; Fig. 4d, f, Supplementary Fig. 6e). These data show that both glutamatergic and GABAergic neurotransmission contributes to the observed GDP-like events.

At the LS, no spontaneous GDP-like events could be recorded (Fig. 4c). To investigate whether the absence of GDPs in day 40 BENOs indicated further maturation of the neuronal networks, we again applied electrical point stimulation. Single pulses of low intensity (100 µA) evoked TTX-sensitive calcium activity at up to 1 mm distance to the stimulation electrode (Fig. 4g). Continuous neuronal network development was demonstrated by a further increase in signal propagation up to 2 mm after stimulation from the same location (*n* = 6 BENOs tested); in some cases, propagation between two BENOs after fusion could be observed (Fig. 4g).

Given that, GDP-like events were suppressed in ES and IS stage BENOs by GABAergic and glutamatergic inhibitors, we assessed whether these neurotransmitters can induce GDP-like events in more mature LS stage BENOs. We found that 10 and 100 µM glutamate could evoke GDP-like events in ES, IS, and LS BENOs (*n* = 3 organoids and 14 ROIs/group; Fig. 4h). In contrast, 10 and 100 µM GABA-evoked GDP-like events only in ES and IS BENOs, but not LS BENOs. These data suggested a developmental switch of GABA from excitatory to inhibitory around differentiation day 40 (*n* = 3 organoids and 14 ROIs/group; Fig. 4h). To verify the inhibitory role of GABA in LS BENOs we measured spontaneous and electrically induced calcium activity under GABA inhibition. GABA inhibition by PTX increased global calcium activity in intensity as well as in frequency, resulting in repetitive synchronized activity reminiscent of epileptic events (*n* = 3 organoids; Fig. 4i). In line with this observation, stimulation-induced calcium release was intensified under GABA inhibition, while glutamatergic network inhibition (CNQX/MK-801) resulted in a marked decrease of signal intensity (*n* = 3 organoids; Fig. 4j). The reduced incidence of GDPs and the characteristic shift from excitatory to inhibitory GABA activity suggest LS BENOs undergo similar staged developmental maturation as observed in the human brain.

### BENOs develop complex network bursts

**Fig. 5:**
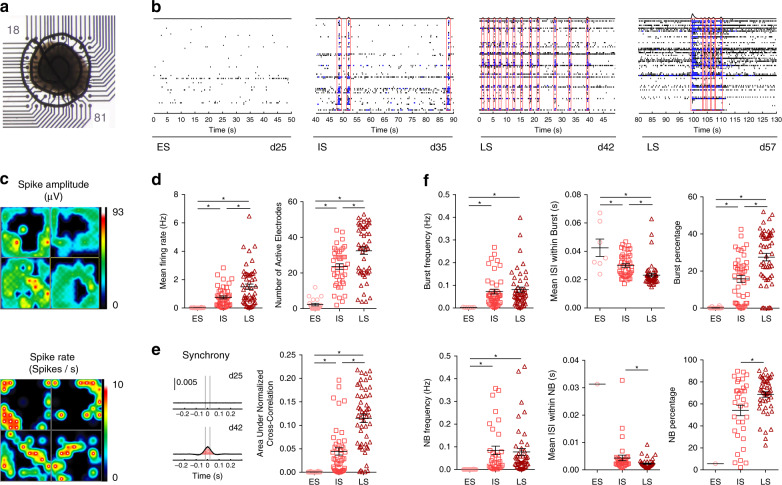
BENOs develop complex network bursts in a course of 2 months. **a** Bright field image of a 300 µm BENO slice mounted on a 64-electrode MEA plate. **b** Representative traces of ES, IS, and LS BENO neuronal networks activity in a 50 s time frame. Note that NB complexity increased with time. **c** Activity heat maps showing spike amplitude (µV) and spike rate (spike/s). **d** General activity parameter such as mean firing rate and number of active electrodes increased with time. **e** Left, representative traces of synchronous activity measured at d25 and d42. Right, the area under the normalized cross-correlation plot (red stripes) was used as a measure of synchrony. **f** Frequency (in Hz), interspike interval (ISI) between burst events (in seconds), and percentage of spikes organized in bursts (in % or total spikes) of and frequency of NB (in Hz). Every dot represents the result of a 10 min measurement of a single BENO. BENO activity was measured daily and results were pooled in ES, IS and LS BENO groups according to the respectively defined time window (ES: <d25; IS: d25 to d40; LS: d40 to d65 of BENO culture). **d**–**f** *n* = 4 organoids, 2 independent experiments. *p < 0.05, one-way ANOVA with Tukey’s multiple comparisons post hoc test. In case of Mean ISI within NB and NB percentage in (**f**) *p < 0.05, unpaired two-tailed Student’s *t* test. Data are presented as mean values ± SEM.

Spatiotemporal neuronal network organization was assessed in d20 BENOs (*n* = 4) over 40 days using multi-electrode arrays (MEAs) to monitor individual network activity (Fig. 5a). ES-BENOs showed sparse electrical activity, which slowly organized in neuronal bursts as well as synchronous activity between neurons in different areas of the organoids, known as network bursts (NB). During the IS, the number of individual bursts and NB increased; in LS BENOs bursts organized in complex synchronous events (Fig. 5b, Supplementary Fig. 7). A heat map of spike amplitude (µV) and spike rate (spike/sec) revealed the regional connectivity of the neurons (Fig. 5c). The mean firing rate and the number of active electrodes significantly increased during the different developmental network stages (LS: 1.5 ± 0.18 Hz and 33 ± 2%; Fig. 5d, Supplementary Fig. 8a). Moreover, there was a dramatic increase in synchronous firing in different regions of the BENO depicted by the area under the normalized cross-correlation graph (ES: 0, IS: 0.04 ± 0.01, LS: 0.12 ± 0.01; Fig. 5e). This was in line with the concomitant increase of the number of spikes participating in bursts and NB (Fig. 5f, Supplementary Fig. 8b,c). Since GABA in BENOs switched from excitatory to inhibitory between IS and LS, we compared other properties at these two stages. Although the burst and NB frequency was unchanged (0.1 Hz) the mean interspike interval (ISI) between burst or NB significantly decreased in LS BENOs to 20 ms and 2 ms, respectively (Fig. 5f). In addition, the NB duration significantly increased with time (Supplementary Fig. 8e). These data demonstrate accelerated formation of complex networks of excitatory and inhibitory neurons in BENOs.

### BENOs present electrically induced neuronal plasticity

**Fig. 6:**
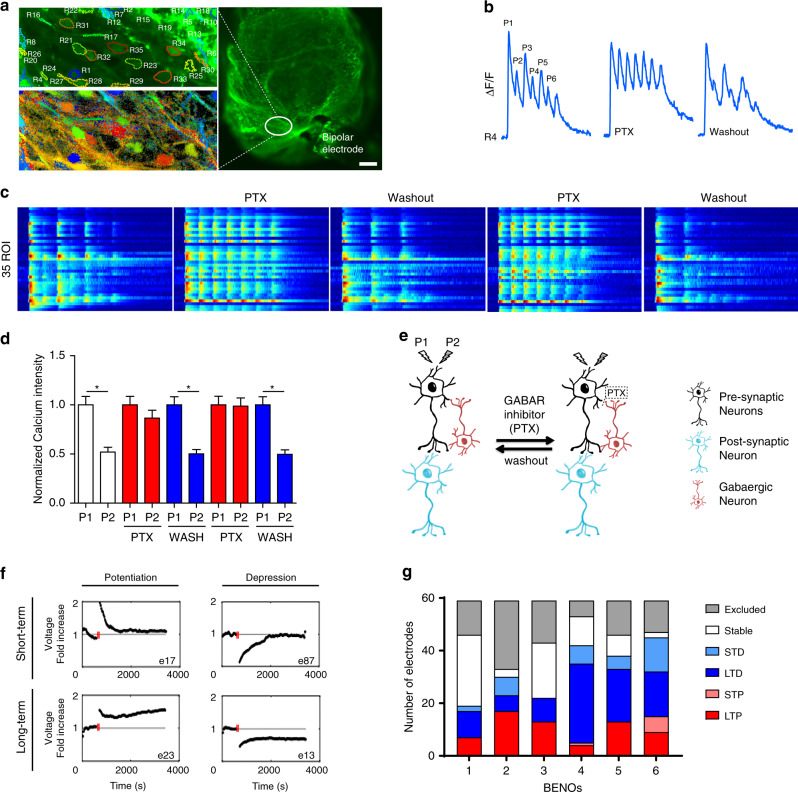
Long-term and short-term neuronal plasticity in BENOs. **a** Right, overview image of a BENO d45 stimulated by a bipolar electrode. Scale bar: 200 µm. Left, close-up view depicting 35 ROIs and the respective active correlation map (same color indicates synchronized activity in network of excitable neurons). Repetition of this experiment on another BENO on d49 is presented in Supplementary Fig. 9b. **b** representative trace showing paired-pulse depression (PPD) between 2 consecutive pulses (P1-P2, P3-P4, P5-P6). PPD was alleviated by PTX and restored upon washout. **c** Heat map of 35 ROIs recorded from a representative BENO showing PPD. **d** Statistical analysis of PPD - calcium activity of ROIs under normal conditions and repetitive treatments with PTX; n = 35 ROIs; in all comparisons *p < 0.0001; unpaired two-tailed Student’s *t* test. **e** Schematic illustrating a simplified neuronal network with the mechanism underlying PPD. P1 activates the presynaptic neuron, which in turn activates a postsynaptic neuron and a GABAergic inhibitory neuron. As a result, the GABAergic neuron releases GABA, which binds to GABA A receptors in the presynaptic neurons resulting in inhibition and consequently decrease in calcium influx. Blockade of GABA receptors by PTX uncouples the network and alleviates the PPD effect. **f** Examples for recordings of short- and long-term potentiation and depression from individual MEA electrodes (**e** refer to activity heat map in Supplementary Fig. 9c, BENO 6), indicated by transient or sustained voltage alterations. HFS is indicated by three red lines. **g** Summary of LTP, LTD, STP, and STD recordings after HFS from six BENO slices (obtained from 6 individual BENOs labeled as 1–6 from 3 independent experiments). Note that each MEA enabled recordings from 59 electrodes; one electrode served as reference. Stable indicates neuronal signal recordings without depression or potentiation. Electrodes without signals are indicated in gray. Data are presented as mean values ± SEM.

Since three of the five most significant GO groups upregulated in BENOs were associated with synaptic plasticity and long-term synaptic potentiation (GO:0048167; GO:0060291 GO:0048168, Supplementary Data 1), we next assessed BENO neuronal plasticity by investigating paired-pulse depression (PPD) as well as short- and long-term potentiation and depression (STP/D and LTD/P). To test for PPD, we stimulated LS BENOS by a bipolar electrode and observed calcium activity of neurons located 200 µm away from the stimulation electrode (Fig. 6a). Paired-pulse stimulation resulted in depression of calcium activity of every second pulse (Fig. 6b–d). Since one of the mechanisms underlying PPD observed in the hippocampus is mediated by presynaptic GABA release^21^, we repeated the stimulation under GABA-receptor blockade with PTX. PPD was alleviated by PTX and re-appeared upon washout, providing evidence for presynaptic GABA release involvement in the observed PPD (Fig. 6b–d, Supplementary Fig. 9a, b depicts detailed traces from two independent BENOs). Another typical form of plasticity observed in the hippocampus and associated with learning^22^ is potentiation or depression of neuronal signal in response to HFS^22^. To test whether BENOs contain neurons which can demonstrate LTP/D, STP/D as signs for plasticity, 300 µm slices (from *n* = 6 BENOs) were placed on MEAs for monitoring of electrical activity (Supplementary Fig. 9c). BENOs were stimulated with single pulses (100 µA, 100 µs pulse width, 30 s inter-pulse interval) until signals were stable and then three pulses of HFS (60 µA, 100 Hz) were delivered. After HFS, electrical signals were recorded for 1 h. Electrodes with a >15% signal increase from baseline indicated potentiation; electrodes with >15% signal decrease from baseline indicated depression (representative traces in Fig. 6f). Evidence for potentiation and depression could be obtained in all investigated BENOs (*n* = 6; Fig. 6g), demonstrating that plasticity is a fundamental property of maturing BENOs.

## Discussion

Until recently, studies on human brain development were limited to rare human fetal brain material^23^. Emerging organoid technologies provide human neuronal cell culture models, which recapitulate in some aspects embryonic brain with cortical layer development^2,7,24^. These 3-dimensional cultures typically rely on neuronal induction in pluripotent stem cell cultures, i.e., typically human embryonic or induced pluripotent stem cell cultures, and subsequent embedding of the differentiated neuronal progenitors in laminin-rich Matrigel™^1–3,5,6,8^, which is well known for being supportive in neuronal cell culture^25^. Alternative cell aggregation models, referred to as cortical spheroids, are developed without the need for the addition of a matrix^4,7,9^. Organoid and spheroid models have been extensively studied as to cell content^1–8^ and directed to develop into distinct brain structures^4–6^. Electrophysiological analyses documented consistently that individual neurons in organoids/spheroids maintain their autonomous function^7,8^. Network formation, being essential for complex brain function, has not yet been characterized in detail in brain organoids, but appears to occur in some models after 9 months^8^. In a recent elegant spheroid study, network oscillations were demonstrated after 7 months^9^. Here, we demonstrate complex network function of the developing (GDP) and the more mature (LTP) human brain after 2 months of BENO culture. Evidence for plasticity underlying learning and memory are in our view particularly exciting as the basis for future exploitation of BENOs in drug development targeting the underlying mechanisms.

BENO development can be divided in four distinct stages: (1) d3–15 commitment to NPC, (2) d15–28 neurogenesis, (3) from d28 onwards neuronal maturation, which is (4) supported by gliogenesis from day 50 onwards. These stages appear to resemble fetal brain development, where neurogenesis precedes gliogenesis. During gliogenesis astrocytes, outer radial glia, and oligodendrocytes developed, with concomitant synaptic maturation, in line with in vivo data^26^. It is important to note that BENOs after d90 contained increasing numbers of myelinated axons comparable to published protocols designed to support cortical spheroid myelination^27^.

Synaptogenesis coincides during development with neuronal connectivity and network formation. To test the hypothesis that BENOs may serve as a model to study and understand neuronal network formation and maturation, we utilized calcium imaging and MEA analyses for longitudinal studies and found similar network activity dynamics in BENOs as reported for the fetal brain. Studies in mouse and human fetal brain, showed that a hallmark of neuronal network development is the presence of GDPs^11^. The TTX-sensitive GDPs are GABA and glutamate dependent^11^ and have been shown to be initiated by pioneer GABAergic somatostatin (SST) positive cells of the hippocampus known as hub neurons^12^. During fetal development GDP frequency initially increases, then decreases and then it ceases completely in early postnatal life^13–15,28^. Similar to the fetal brain, BENOs present TTX-sensitive GDP-like events, which are GABA and glutamate dependent. The GDP-like event frequency increases between day 25 and 40, but significantly decreases in later stages. After culture day 40, no GDP-like events were recorded. However, we cannot exclude that GDPs may still occur at very low frequency. BENO transcriptome profiling showed enrichment of hippocampal neuronal markers, including SST, between days 28 and 40. Future experiments based on genetic tracing of SST^pos^ neurons in combination with calcium imaging may help to identify the specific mechanisms underlying GDP, which includes the investigation whether GABA^pos^ SST^pos^ hub neurons^12^ initiate the GDP-like events. Although we did not systematically examine BENOs for parvalbumin positive GABAergic neurons, the transcriptomic data show a small increase at d60, thus suggesting that it is a matter of time for this population to emerge. A more systematic analysis, including single cell sequencing in d90 organoids should be performed in the future in order to fully appreciate the dynamics of BENO cellular complexity.

Another hallmark of neuronal network maturation is the switch of excitatory to inhibitory GABAergic neurotransmission^29^. Interestingly, the disappearance of GDP-like events in BENOs coincided with the switch of GABA from excitatory to inhibitory. In contrast to BENOs, 2D cultures of iPSC-derived forebrain neurons show GDP-like events at low frequency only after prolonged culture (d58 in 2D vs d22 in ES BENOs) and a peak of GDP-activity by d70 in 2D culture (vs d35 in IS BENO culture). Reduction in GDP was reported after d100 in 2D (vs d40 LS BENOs)^17^. The 2D forebrain cultures contained few GABAergic neurons and thus their contribution to GDP-like events could not be demonstrated^17^. Although no GDP events were previously demonstrated in an organoid level, Sakaguchi et al. showed that 109d-old neurons (deriving from dissociated brain organoids) exhibit local calcium networks bursts^30^. They further revealed that these areas contained both glutamatergic and GABAergic neurons. These data are in agreement with our findings and underline the importance of inhibitory neurons for comprehensive network formation. The expedited neuronal development in BENOs was further evidenced by the observation of features indicating network complexity after 2 months in culture, whereas similar features have been reported recently in other brain organoid models after 7 months in culture^9^. In both studies the network complexity increased in the presence of inhibitory GABAergic neurons (2 months in BENOs vs 6-10 months in cortical spheroids^9^). Our data, in line with the recent data from the Muotri group^9^ underscore the requirement of inhibitory GABAergic neurotransmission for neuronal network maturation in brain organoids.

Another hallmark of network function is neuronal plasticity. Our study identified phenomena associated with neuronal plasticity being present in BENOs, such as PPD and STP/D and LTP/D. PPD, a typical form of plasticity observed in the hippocampus, is explained by presynaptic mechanisms, such as neurotransmitter depletion or postsynaptic mechanisms^21^. In BENOs, the observed PPD was mediated by postsynaptic inhibitory GABAergic neurons since GABA-A receptor blockade could completely abrogate this phenomenon. These data demonstrate the importance of interneurons in plasticity and complex network function. Interestingly, evolutionary studies have shown that the amount of interneurons in primates is much higher than rodents (40% vs 20% of cortical neurons respectively, see review)^31^. LTP is a typical electrophysiological feature of plasticity, which is observed in the hippocampus and is the underlying mechanism for learning and memory. In mouse hippocampal slices LTP is measured after HFS^32^. Similarly, 1 h upon HFS, electrical network activity in BENOs demonstrated long- and short-term potentiation or depression, providing evidence for stimulus entrained plasticity in a human brain organoid model.

Although, brain organoids represent useful models to study brain development, cellular heterogeneity and limited network function are considered key caveats. Transcriptional and protein profiles in BENOs suggested the presence of glutamatergic, GABAergic, catecholaminergic, and serotinergic neurons as well as myelinating (oligodendrocytes) and non-myelinating (astrocytes) glia. BENOs traverse through distinct phases of development with primitive network function (GDP) and more advanced network function (PPD and LTP/D), which requires the interplay of excitatory (glutamatergic) and inhibitory (GABAergic) neurons. Our data suggest that the use of brain organoids in developmental or drug discovery studies has to carefully consider their developmental stage to address specific pathophysiologically relevant questions. The development of a natural cellular composition (e.g., excitatory and inhibitory neurons) and the definition of the state of electrophysiological maturation (day 25 vs 50 in BENOs with little and consistent network activity, respectively) are important factors when designing drug discovery experiments, for example in the context of epilepsy.

## Methods

### BENO generation (protocol 5)

The GMP hiPS line (TC1133, *Lonza*)^33^ and an in-house generated line (hiPS-G1)^34^ were used for BENO generation. The BENO differentiation protocol was established using the hiPS-G1 reference line. Immunofluorescence analyses and the functional data were replicated in both lines to test for protocol robustness. One day prior to neuronal differentiation, a 1:1 mixture of acid-solubilized bovine collagen I (Collagen Solutions) and serum free 2× DMEM (Thermoscientific) was prepared and neutralized by 0.1 M NaOH addition. iPSC suspensions (4500 cells/µl) were prepared in StemMACS™ iPS-Brew XF medium (Miltenyi) complemented with 20 ng/ml of FGF-2 (Miltenyi) and 10 µmol/L Y-27632 (Stemgent). iPSCs were added to the collagen/DMEM mixture to achieve a final concentration of 3,000 cells/µl and 1 mg/ml of collagen I. Thirty microliters of cell-collagen mixtures was aliquoted into 96-well plates (U-bottom, low attachment) and placed in an incubator for 30 min. Upon collagen polymerization, 250 µl of StemMACS™ iPS-Brew XF supplemented with 10 ng/ml FGF and 10 µmol/L Y-27632 were added in each well. From day 0 to 10 BENOs were cultured in neuronal commitment medium (NCM, Basal medium [Neurobasal-A containing 2 mmol/L glutamine, 100 U/ml penicillin, 100 µg/ml streptomycin, 2% B27, 1% N2 supplement, 200 µmol/L ascorbic acid] complemented with 10 µmol/L SB 431542 [Tocris], 50 ng/ml noggin [R&D systems] and 1 µmol/L RA [Sigma]). On day 3, BENOs were transferred into six-well plates (10 BENOs/well). From day 10 to 15 BENOs were cultured in neural progenitor expansion medium (NPEM, Basal medium complemented with 10 ng/ml FGF-2 and 5 ng/ml TGFB1 [Peprotech]). Finally, from day 15 to 28 BENOs were cultured in Neural Differentiation Medium (Basal medium complemented with 2.5 µmol/L DAPT [Tocris] and 5 ng/ml TGFB1). From day 29 and until the day of analysis, BENOs were cultured in Basal medium. Medium change was performed every second day. BENO preparation protocols 1–4 were modified as depicted in Fig. 1a. Note that large quantities of BENOs can be generated in parallel with highly reproducible morphology (Supplementary Fig. 1e). A step-by-step protocol describing the generation of BENOs can be obtained from Nature Protocol Exchange^35^.

### Calcium imaging

For calcium analysis, whole BENOs were loaded for 15–30 min with 1 µg/ml Fluo-8-AM in carbogenated artificial cerebrospinal fluid (ACSF) buffer (mmol/L: 26 NaHCO_3_, 10 dextrose, 1 MgSO_4_·7H_2_O, 1.25 NaH_2_PO_4_, 2.5 KCl, 126 NaCl, 2 CaCl_2_), pH 7.3. Calcium imaging was performed on a confocal microscope (Zeiss LSM 780 equipped with ZEN 2010 software), while BENOs were continuously perfused with ACSF and the temperature was maintained at 37 °C. Fluorescence was recorded at 2–5 Hz whilst electrical stimulation was given at a defined time with five pulses (50 µs, 50–100 µA) with ISI 75 ms every 2 s. Action potential mediated calcium activity was blocked by 1 µmol/L TTX (final concentration) for 1 min and reperfusion with ACSF. Similarly, GABA-B receptors were blocked by 330 µmol/L Saclofen (Tocris), GABA-A and -C receptors were blocked by 58 µmol/L Picrotoxin (PTX; Sigma), NMDR by 0.2 µmol/L (+)-MK-801 hydrogen maleate (Sigma) and AMPAR by 15 µmol/L CNQX (Sigma). Activation of the GABAergic or the glutamatergic network was achieved by 10 µmol/L GABA (Sigma) or 10 µmol/L Glutamic acid (Sigma) respectively. Automated synchronization detection was performed using Matlab R2012a (The Math Works, USA).

### Electrophysiology analysis

Na and K currents were recorded by conventional whole-cell patch-clamping. BENO slices were stained with Fluo-8-AM as described above. Ca-activity was visualized with an upright microscope (Zeiss LSM 780 equipped with ZEN 2010 software). Patch pipettes (2–4 MΩ) were pulled from borosilicate glass (1.6 mm outside diameter) and filled with intracellular solution containing (in mmol/L): 135 HMeSO_4_, 10 KCl, 1 EGTA, 10 HEPES, 2 MgCL_2_, 2 ATP-Na2, 0.1 GTP-Na, 6.6 Phosphocreatine-Na2, with pH adjusted to 7.2–7.4 by KOH, and osmolarity adjusted to 290–295 mOsm. Membrane currents were elicited by a series of depolarizing pulses between −60 mV and +50 mV, in 10 mV increments, from a holding potential of −70 mV. An EPC-9 patch-clamp amplifier equipped with Patchmaster software (HEKA Electronics, Germany) was used for data acquisition. Liquid-junction potentials, leak currents (P/n protocol), fast and slow capacitances^36,37^, and series resistances were corrected on-line. The data were sampled at 20 kHz and filtered at 10 kHz (four-pole Bessel) and 5.9 kHz (three-pole Bessel). Data were stored and exported to Matlab (The Math Works, USA) for subsequent analyses.

### MEA recording

Six-well plates with 64 platinum microelectrodes arrays per well (MEA; 0.04 MΩ/microelectrode, 30 µm microelectrode diameter, 200 µm spacing) were coated with Matrigel™ 1:120 diluted in PBS for 1 h at room temperature prior seeding. One or two slices (thickness: 300 µm) were placed into a MEA well and immobilized by a concentrically coiled tungsten ring. 10–15 min recordings/2 h were performed every other day for up to 60 days using a Maestro pro MEA system (Axion Biosystems). Data recordings were automatically scheduled with the Axion Software (AxIS Navigator) using the manufacturer’s Spontaneous Neural Configuration. Data analysis was performed using the manufacturer’s standalone tools, Neural Metric Tool, and AxIS Metric Plotting Tool (Axion Biosystems). The spike detecting threshold was set to 5.5 standard deviations, and the electrodes that detected at least 5 spikes per min were classified as active. Spike bursts were identified using an ISI threshold requiring a minimum number of five spikes with a maximum ISI of 100 ms. NB were identified by an envelope algorithm with a threshold of 1.25, minimum IBI interval of 100 ms, 75% burst inclusion with a minimum of 10% active electrodes. Firing synchrony was estimated by the area under the normalized synchrony cross-correlogram for a time window of 20 ms.

For potentiation measurements, multi-channel fEPSP recordings were performed with a MEA2100 device (MC_Rack 3.2.1.0 software, Multi Channel Systems, Reutlingen, FRG). To reduce noise, the bath was grounded via an extra custom-made Ag/AgCl electrode attached to the MEA amplifier ground socket. LTP was induced by 3 trains of HFS (20 pulses 100 Hz, 50 µs, 100 µA) in 20 s interval. Prior to HFS, neurons were stimulated by single pulses (100 µA, 50 µs pulse width, 30 s inter-pulse interval). After stimulation the same protocol was performed for 1 h in order to quantify differences in synaptic strength.

### Wholemount immunofluorescence (WmIF)

BENOs were fixed with 4% formaldehyde solution (Histofix, Carlroth) for 2 h at 4 °C. Subsequently, they were washed twice with PBS and blocked for 30 min at 4 °C with staining buffer (StB; 5% FBS, 1% BSA, 0,5% Triton X-100 in PBS). BENOs were incubated with primary antibodies diluted in StB for 2 days at 4 °C (100 µl/BENO). Upon washing with StB for 6–8 h, BENOs were incubated with secondary antibodies and Hoechst 33342 (Sigma) for another 2 days at 4 °C. After StB washings for a total of 6–8 h BENOs were mounted on glass coverslips. An antibody list with respective dilutions is provided in Supplementary Data 2. WmIF was visualized using confocal imaging performed on a Zeiss LSM 710 confocal microscope equipped with ZEN 2010 software.

### RNA extraction and quantitative PCR

RNA was extracted from BENOs with the NucleoSpin RNA isolation kit (Macherey‐Nagel), according to the manufacturer’s instructions. Reverse transcription was performed using Oligo(dT)_20_ primer (Eurofins Genomics), dNTP mix and M‐MLV reverse transcriptase (Promega). For qPCR analysis SYBR Green (Promega and Eurogentec) and a 7900 HT Fast real‐time PCR system (Applied Biosystems) were used. qPCR data were collected and analyzed by SDS2.4 software (Applied Biosystems). Primer sequences are provided in Supplementary Data 3.

### RNA sequencing

RNA was isolated using the Macherey-Nagel RNA isolation kit (cat. no. 740955). RNA integrity was verified by Agilent Bioanalyzer 2100. The cDNA library was prepared from 100 ng of total RNA, by TruSeq Stranded Total RNA Sample Prep (Illumina) according to the manufacturer’s instructions. Briefly, total RNA was depleted from ribosomal RNA by magnetic bead separation. Ribodepleted RNA was fragmented and first strand synthesis was performed. Second strand synthesis was performed using dUTP so that at the PCR amplification step only the first strand was amplified. Prepared libraries were quantified using the Qubit High Sensitivity Assay (Invitrogen), the size distribution was controlled using Bioanalyzer 2100 and sequencing was performed using HiSeq2000 (SR 50 bp) according to the manufacturer’s instructions. Bcl files were demultiplexed and converted to fastq using fastq2bcl. Fastq files were mapped using TopHat (v2.1.1) and fragments per kilobase of transcript per million (FPKM) calculated using Cufflinks (v2.2.1)^38^. Only protein coding transcripts were considered for further analysis. Human genome annotation used was GRCh38.87. All genes with FPKM < 1 were omitted for the respective data sets for Fig. 2. Differential gene expression was performed using Cuffdiff^39^. GO analysis was performed by ClueGo plugin (v2.5.1)^40^ in cytoscape (v3.6.1)^41^. The visualization of data was done in R.

### Statistical testing

All data are displayed as mean ± standard error of mean (SEM). The investigated sample number is provided as *n*. Statistical differences between two groups were tested by two-tailed unpaired Student’s *t* tests. In case of three and more groups, one-way or two-way ANOVA with appropriate post-hoc testing was performed. The performed statistical tests are specified in the respective figure legends. Statistical significance was assumed if *p* < 0.05. RNAseq data were corrected according to Benjamini and Hochberg^42^. For statistical analyses and graphical display of the data Graph Pad Prism (GraphPad Software) was used.

### Reporting summary

Further information on research design is available in the Nature Research Reporting Summary linked to this article.

## Supplementary information

Supplementary Information

Description of Additional Supplementary Files

Supplementary Data 1

Supplementary Movie 1

Supplementary Movie 2

Supplementary Movie 3

Reporting Summary

## Data Availability

The gene array data sets generated in this work have been deposited in Gene Expression Omnibus under the accession number GSE139101. The data that support the findings of this study are available from the corresponding author upon reasonable request.
