# Supplementary material for: Developmental GABA polarity switch and neuronal plasticity in Bioengineered Neuronal Organoids
Source: Nat Commun. 2020 Jul 29;11:3791. doi: 10.1038/s41467-020-17521-w (PMC7391775; doi:10.1038/s41467-020-17521-w)
Supplement: Supplementary file 1 — Supplementary Information [file 41467_2020_17521_MOESM1_ESM.pdf]

Supplementary Information

Developmental GABA polarity switch and neuronal plasticity in Bioengineered Neuronal Organoids

Zafeiriou et al.

Supplementary Figures

Supplementary Figure 1. Effect of variable neuronal induction duration, FGF and TGFβ1 treatment in BENO generation.

Supplementary Figure 2. Transcript analysis of developing BENOs by qPCR from d-1 to d40 under the tested protocols.

Supplementary Figure 3. Transcriptome analysis of BENO development in the course of 2 months.

Supplementary Figure 4. WmIF analysis shows functional excitatory neurons in d40 BENOs.

Supplementary Figure 5. Myelination in BENOs.

Supplementary Figure 6. Development of neuronal networks during BENO generation.

Supplementary Figure 7. MEA monitoring of neuronal network development in d20-d57 BENOs.

Supplementary Figure 8. Parameters describing neuronal network activity in d20-d57 BENOs.

Supplementary Figure 9. Network plasticity in BENOS.

Supplementary Tables

Supplementary Table **1**. Detailed antibody list containing respective dilutions for WmIF.

Supplementary Table **2**. Detailed primer sequence list.

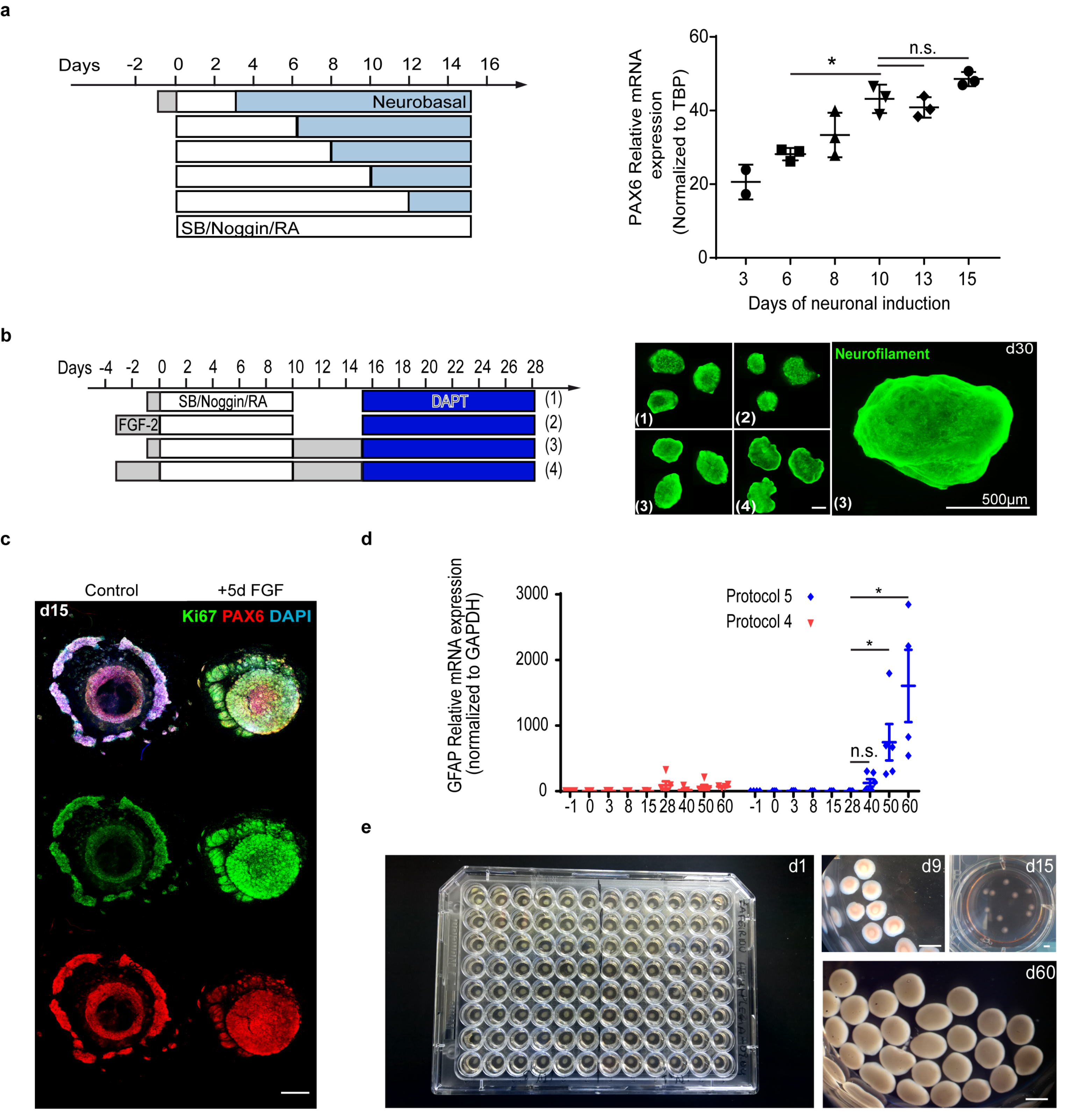

Supplementary Fig. 1| Effect of variable neuronal induction duration, FGF and TGFβ1 treatment in BENO generation. a, Schematic overview of the tested neuronal induction protocols and PAX6 transcript expression in the respectively derived d15 BENOs. Neuronal induction by dual SMAD inhibition (Noggin/SB) was performed for 3, 6, 8, 10 12 or 15 days. After termination of dual SMAD inhibition all BENOs were cultured until day 15 in basal medium and subjected to RNA isolation for qPCR. TATA sequence binding protein (TBP) was used as housekeeping gene; n=3 / time point. *$p<0.05$, one-way ANOVA with Sidak's multiple comparisons post hoc test. **b**, Effect of FGF2 treatment on neurofilament expression in d30 BENOs. BENOs were treated with 10 ng/ml FGF2 prior neuronal commitment (cond. 2), after (cond. 3) or both (cond. 4) and compared with the untreated control (cond. 1); refer to the schematic for an overview of the differently tested conditions. Conditions 3 and 4 showed similar NF positive axons interconnecting throughout the organoid in contrast to condition 1 and 2. These data suggest that FGF2 addition after neural induction is crucial for BENO generation. **c**, Whole mount immunofluorescence (WmIF) analysis of BENOS at d15 demonstrated proliferating NPCs (PAX6pos / Ki67pos). 5 d treatment of BENOs with FGF2 after neuronal commitment clearly increased the number of proliferating NPCs and supported tissue condensation. Scale bar: 500 µm. d, Transcript levels of GFAP throughout BENO development with (protocol 5; Figure 1a) and without (protocol 4; Figure 1a) TGFβ1. TGFβ1 markedly enhanced gliogenesis after day 30. GAPDH was used as a housekeeping gene. n=3-5/time point, 2 Independent experiments, data are presented as mean values +/- SEM, *$p<0.05$, two-way ANOVA with Sidak's multiple comparisons post hoc test. e, Bright field images showing the morphological homogeneity of BENOs in different days of differentiation (d1, d9, d15, d60). Scale bar: 2 mm.

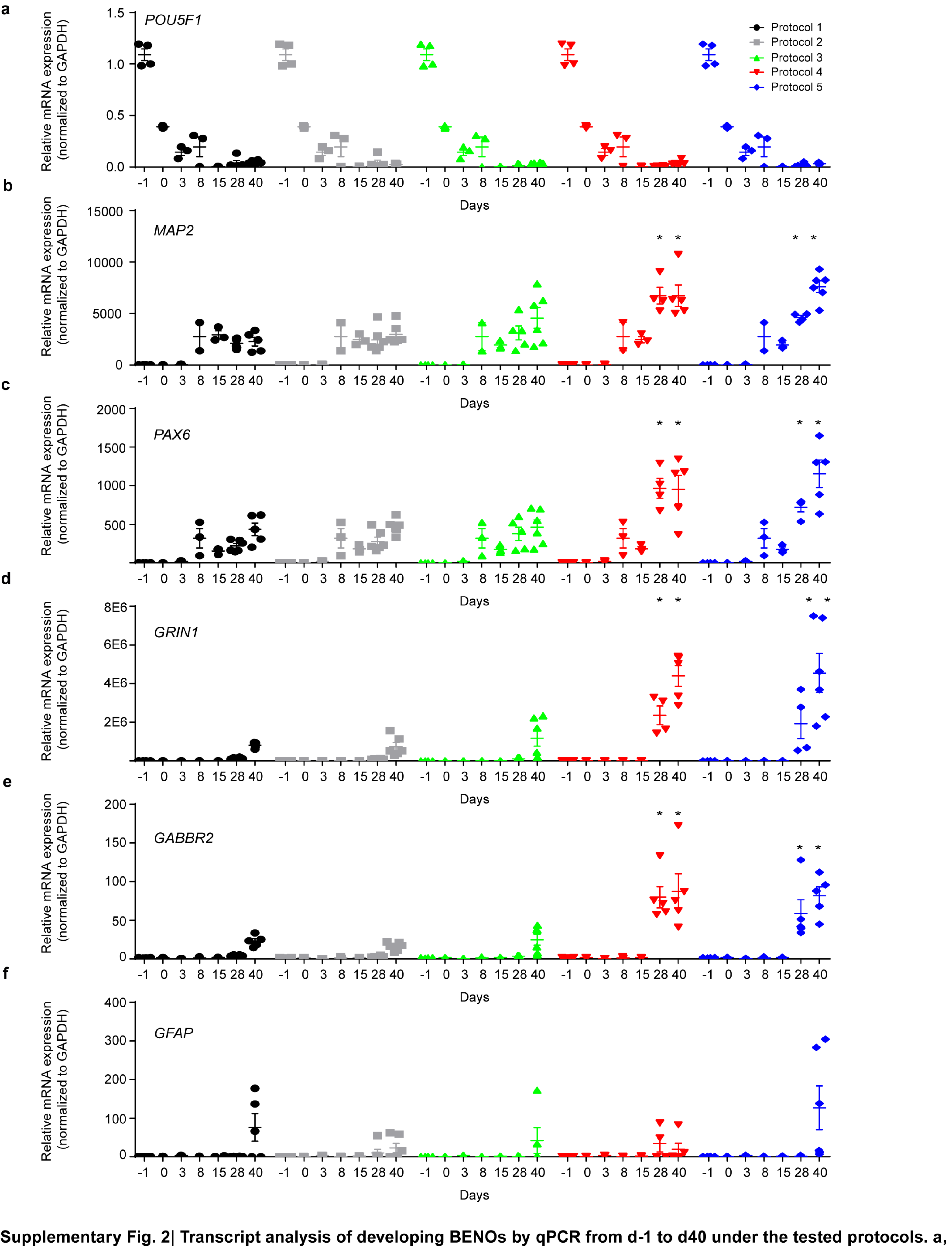

Supplementary Fig. 2| Transcript analysis of developing BENOs by qPCR from d-1 to d40 under the tested protocols. a, Pluripotency marker OCT4 (POU5F1) was diminished by day 15. Neuronal markers **b**, MAP2 and **c**, PAX6 increased from d8 and onwards. **d,** Glutamatergic receptor GRIN1 and **e**, GABAergic receptor GABBR2 transcription were enhanced by DAPT treatment from day 28 and onwards. **f**, Glia marker GFAP was increased at day 40 in the presence of TGFβ1. This figure presents parts of the data shown in Supplementary Fig. 1d. Data were normalized to GAPDH. n=3-5 BENO/time point with the exception of d0 and d3 samples were each biological replicates consisting of 10 organoids, data are presented as mean values +/- SEM. *$p<0.05$, two-way ANOVA with Tukey's multiple comparisons post hoc test.

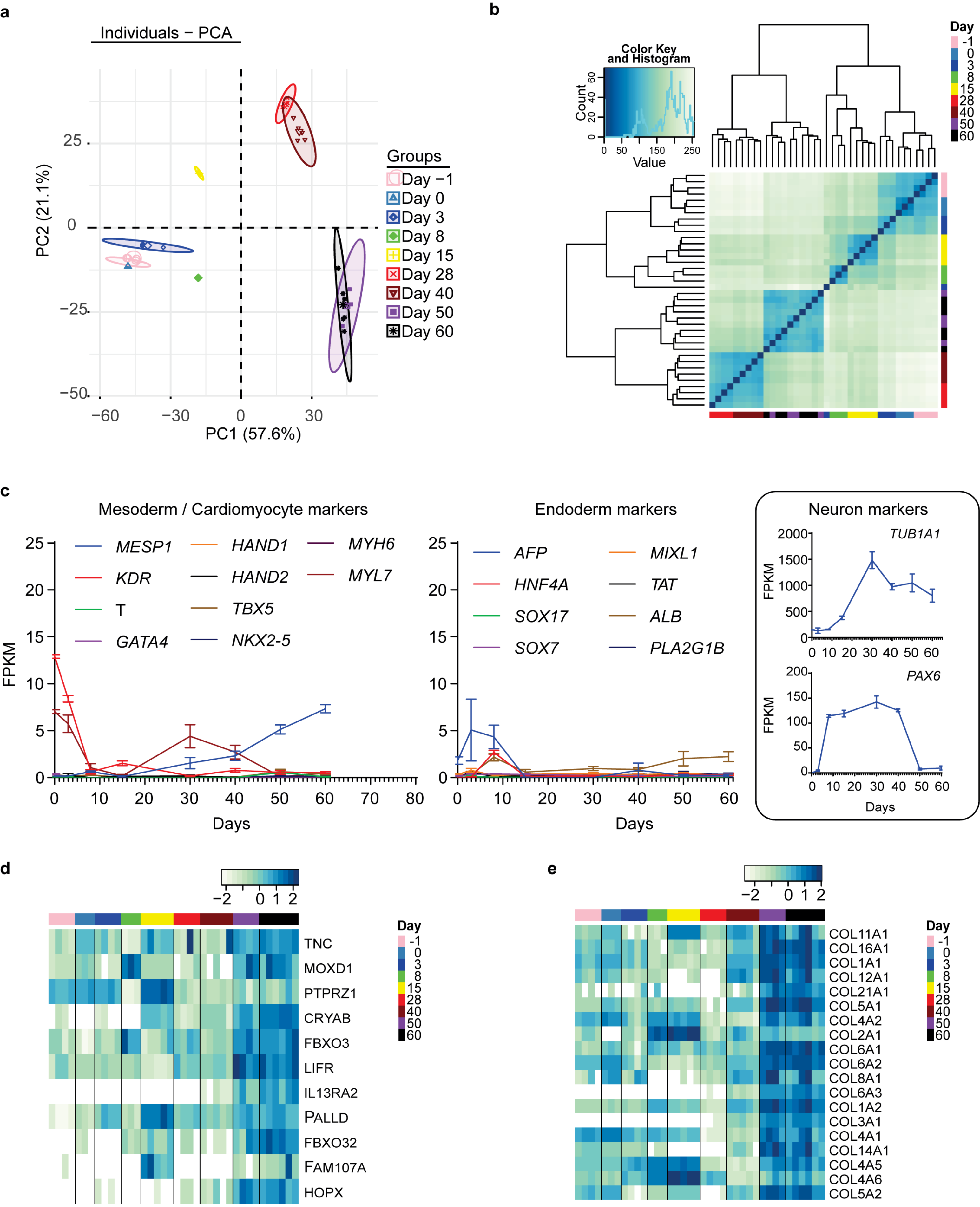

Supplementary Fig. 3| Transcriptome analysis of BENO development in the course of 2 months. a, Principal component analysis and **b**, correlation heat map showing clustering of data obtained from BENOs at: d-1, 0 and 3 (stem cell); d8 and d15 (NPC); of d28, d40 (neurogenesis); and day 50, 60 (gliogenesis). **c**, very low FPKM values for mesodermal and endodermal markers provide evidence for no or negligible contamination of BENOS with non-ectodermal lineages. Structural neuronal markers TUB1A1 as well as transcription factor PAX6 were used as reference for the neuronal component. **d**, Gene expression heat map for markers of outer radial glia during BENO development. **e**, Gene expression heat map of matrix components upregulated during gliogenesis (d50-60).

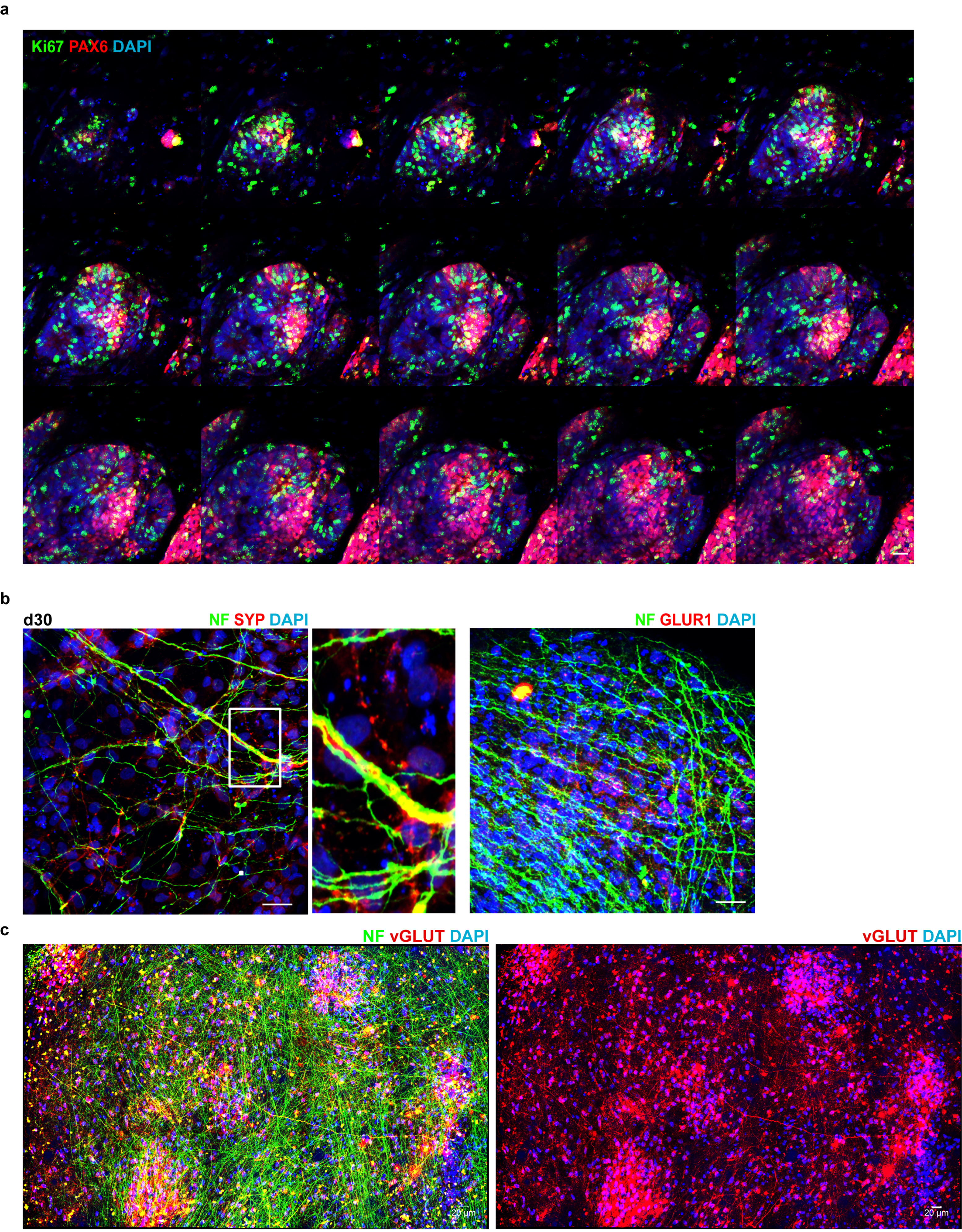

Supplementary Fig. 4| WmIF analysis shows functional excitatory neurons in d40 BENOs. a, The confocal planes of the higher magnification image depicted in Figure 3a are displayed to distinguish proliferating NPCs with nuclear PAX6 and Ki67 signals. Scale bar: 20 µm. **b**, Prominent expression of presynaptic marker synaptopshysin (SYP) and postsynaptic marker GLUR1 suggested the presence of synapses. **c**, Presynaptic marker vGlut demonstrated a high abundance of glutamatergic neurons in BENOs. The expression was localized in the somas as well as in synaptic boutons. All data presented on this figure derive from at least 3 independent experiments with similar results. Scale bar: 20 µm.

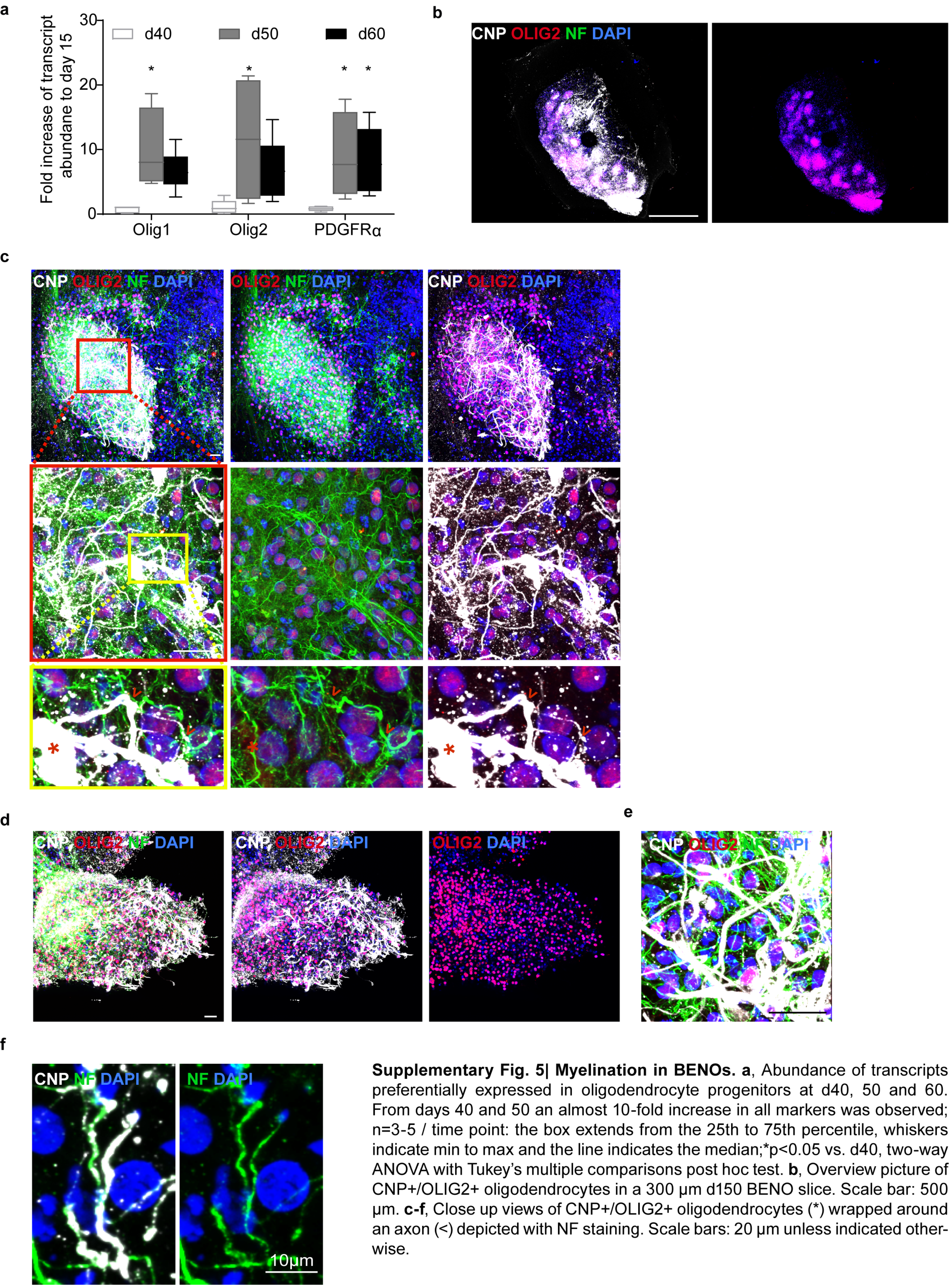

Supplementary Fig. 5| Myelination in BENOs. a, Abundance of transcripts preferentially expressed in oligodendrocyte progenitors at d40, 50 and 60. From days 40 and 50 an almost 10-fold increase in all markers was observed; n=3-5 / time point: the box extends from the 25th to 75th percentile, whiskers indicate min to max and the line indicates the median;*p<0.05 vs. d40, two-way ANOVA with Tukey's multiple comparisons post hoc test. **b**, Overview picture of CNP+/OLIG2+ oligodendrocytes in a 300 µm d150 BENO slice. Scale bar: 500 µm. **c-f**, Close up views of CNP+/OLIG2+ oligodendrocytes (*) wrapped around an axon (<) depicted with NF staining. Scale bars: 20 µm unless indicated otherwise.

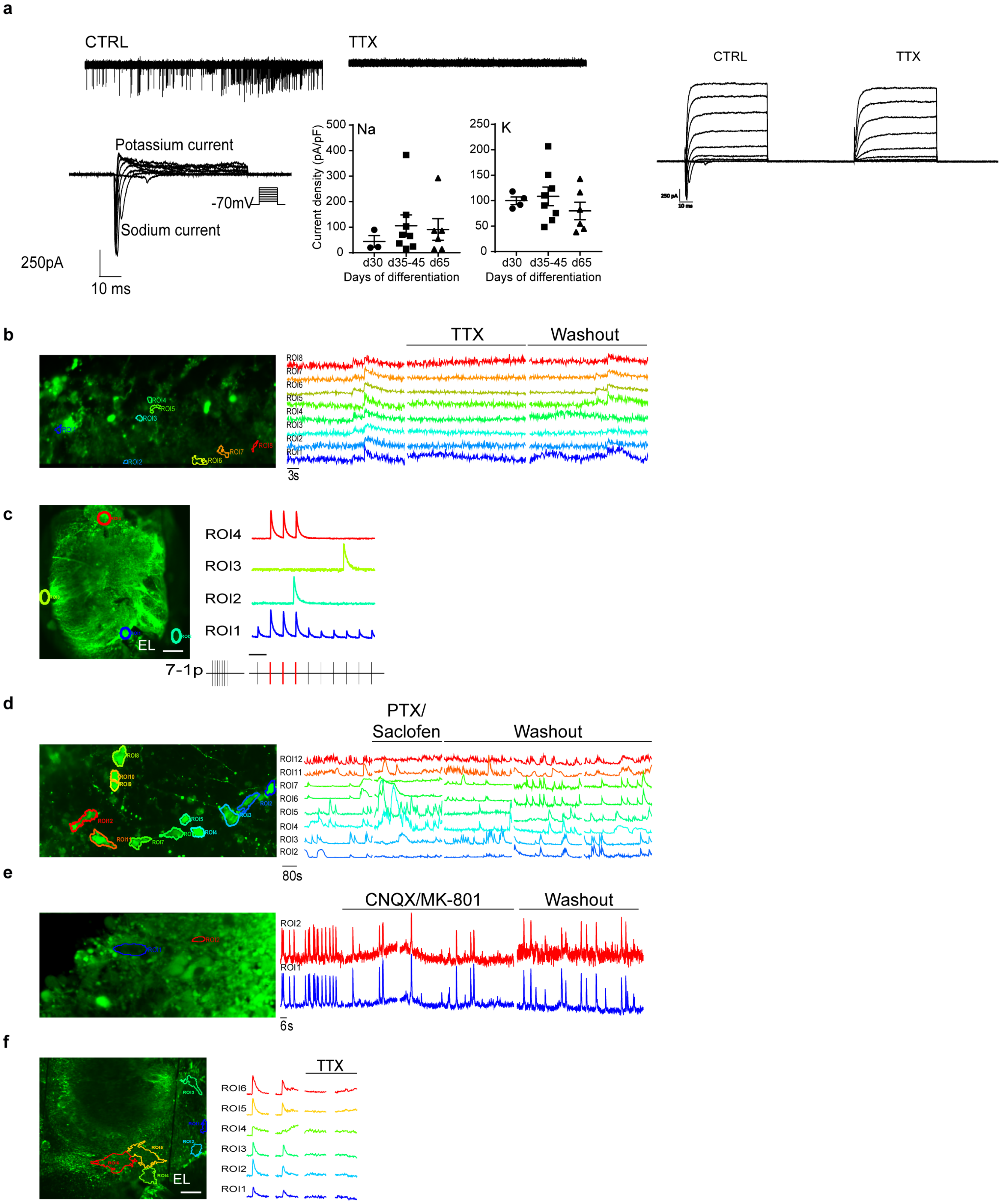

Supplementary Fig. 6| Development of neuronal networks during BENO generation. a, Representative excitatory postsynaptic potential (EPSP) trace of a neuron in d30 BENOs disappears upon TTX treatment. Trace of a typical sodium and potassium current upon whole-cell patch-clamp recording. Right, sodium and potassium current density during different stages of neuronal development (d30, d35-45, d65). Patch recordings are from single neurons (data averaged from 3 independent BENOs/time point +/- SEM). **b**, TTX-sensitive spontaneous activity in 21 days old BENO. **c**, GDP-like events stimulated in d27 BENO by high frequency electrical stimulation (HFS) administered via a bipolar electrode to the indicated region of interest (ROI) 1. Single pulses stimulated the proximal region, but did not propagate to ROI4. ROI2 and 3 show only spontaneous regional GDPs. Stimulation was delivered every 30 s. Single pulses are depicted with black lines and HFS with red. Scale bar: 200 μm. **d, e**, Close-up view showing synchronized calcium activity of individual neurons in d35 BENOs. Traces of four different pairs of synchronous ROIs (2-3, 4-5, 6-7, 11-12) are desynchronized upon GABA block by PTX/Saclofen and re-synchronized upon washout (d). Reduction of spontaneous activity by glutamatergic block by CNQX/MK-801 in two ROIs (e). **f,** TTX-sensitive activity after electrical stimulation of a d21 BENO. Scale bar: 200 μm.

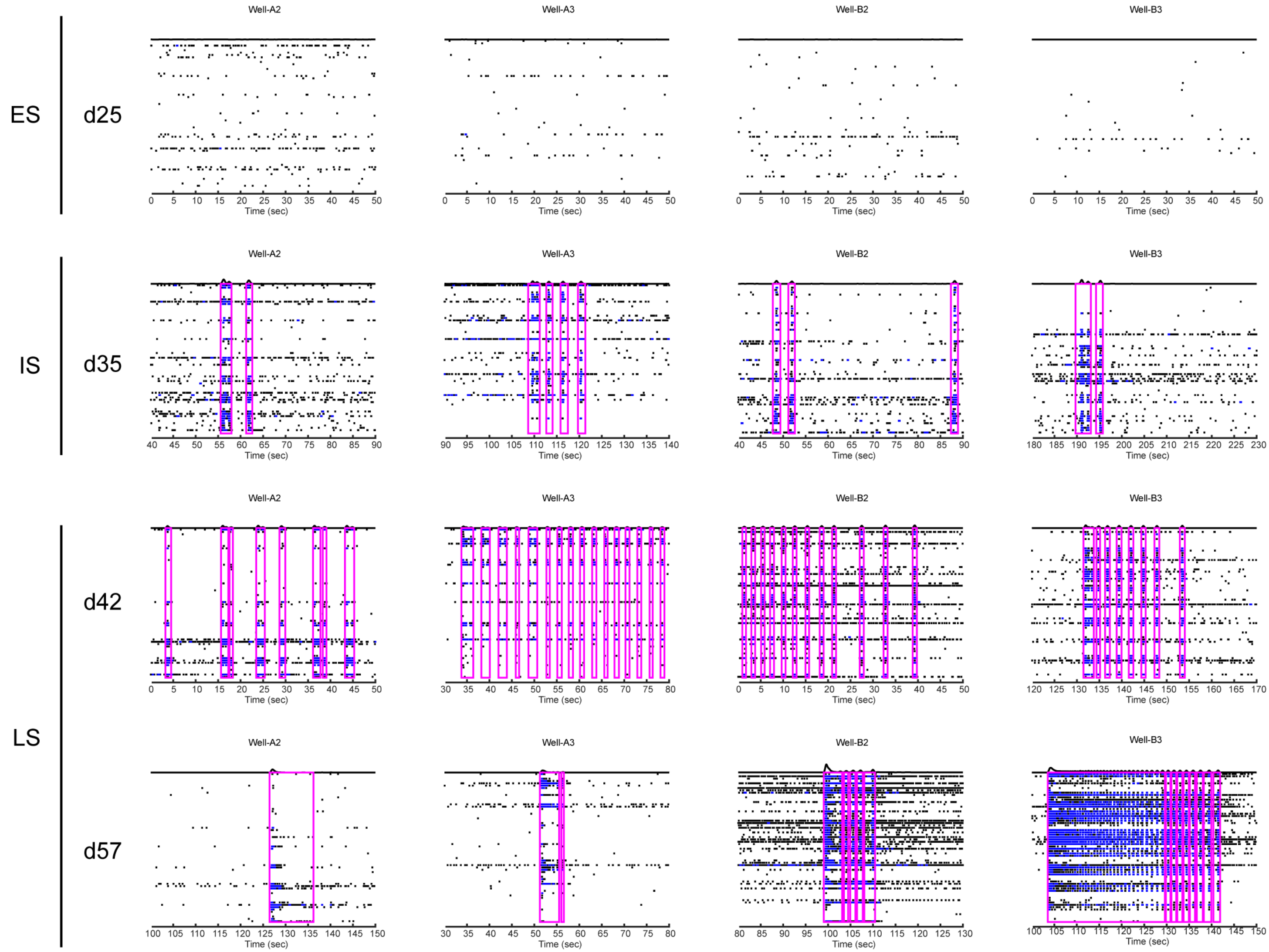

Supplementary Fig. 7| MEA monitoring of neuronal network development in d20-d57 BENOs. Representative raster plots of 4 BENOs at different time points. In the snapshot only 50 sec from 10 min measurements are captured. At d25 no network bursts (NB) were detected; at d35 NBs appeared scarcely; at d42 NBs appeared frequently; at d57 NBs developed complex patterns.

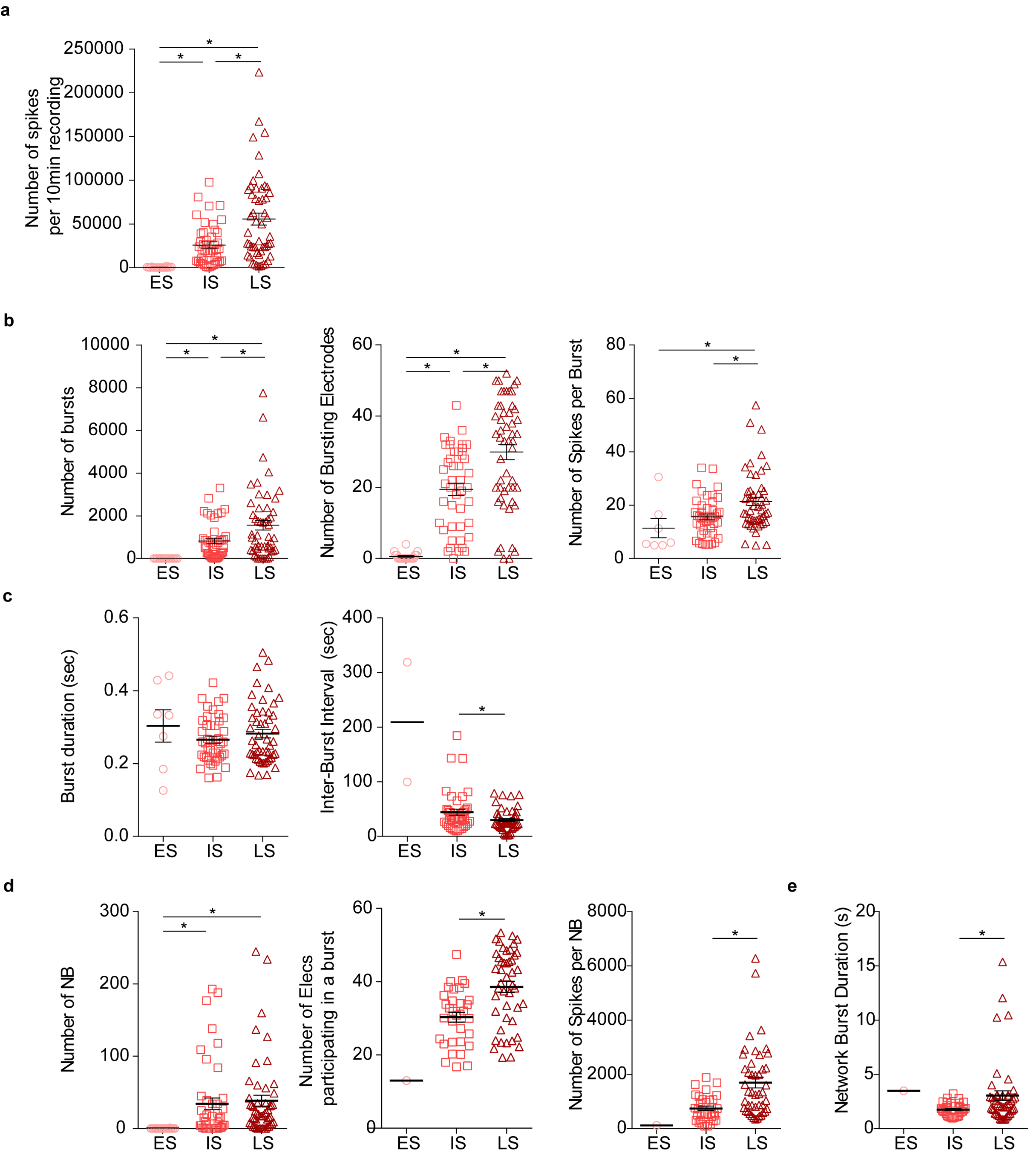

Supplementary Fig. 8| Parameters describing neuronal network activity in d20-d57 BENOs. a, Parameter of general neuronal activity: more than 5,000 spikes per min recording were detected in average in d57, i.e., LS-BENOs. **b**, Parameters describing the organization of spikes in bursts of a single electrode: total number of bursts, the number of bursting electrodes, and the number of spikes in a burst significantly increased over time. **c**, Although burst duration did not increase, the inter-burst interval significantly decreased resulting in an increased burst frequency. **d**, Parameters describing the organization of bursts into network bursts: the total number of NB, the number of electrodes participating in a NB, and the number of spikes in a NB significantly increased over time. e, network burst duration increased over time revealing the complexity of the network at LS BENOs. Data are presented as mean values +/- SEM. *p<0.05 one-way ANOVA with Tukey's multiple comparisons post hoc test (a-c); For parameters where ES BENOs contained less than 3 values, IS and LS BENOs were analysed by unpaired two-tailed Student's t- test*p<0.05. ES: early stage BENOs; IS: intermediate stage BENOs; LS: late stage BENOs.

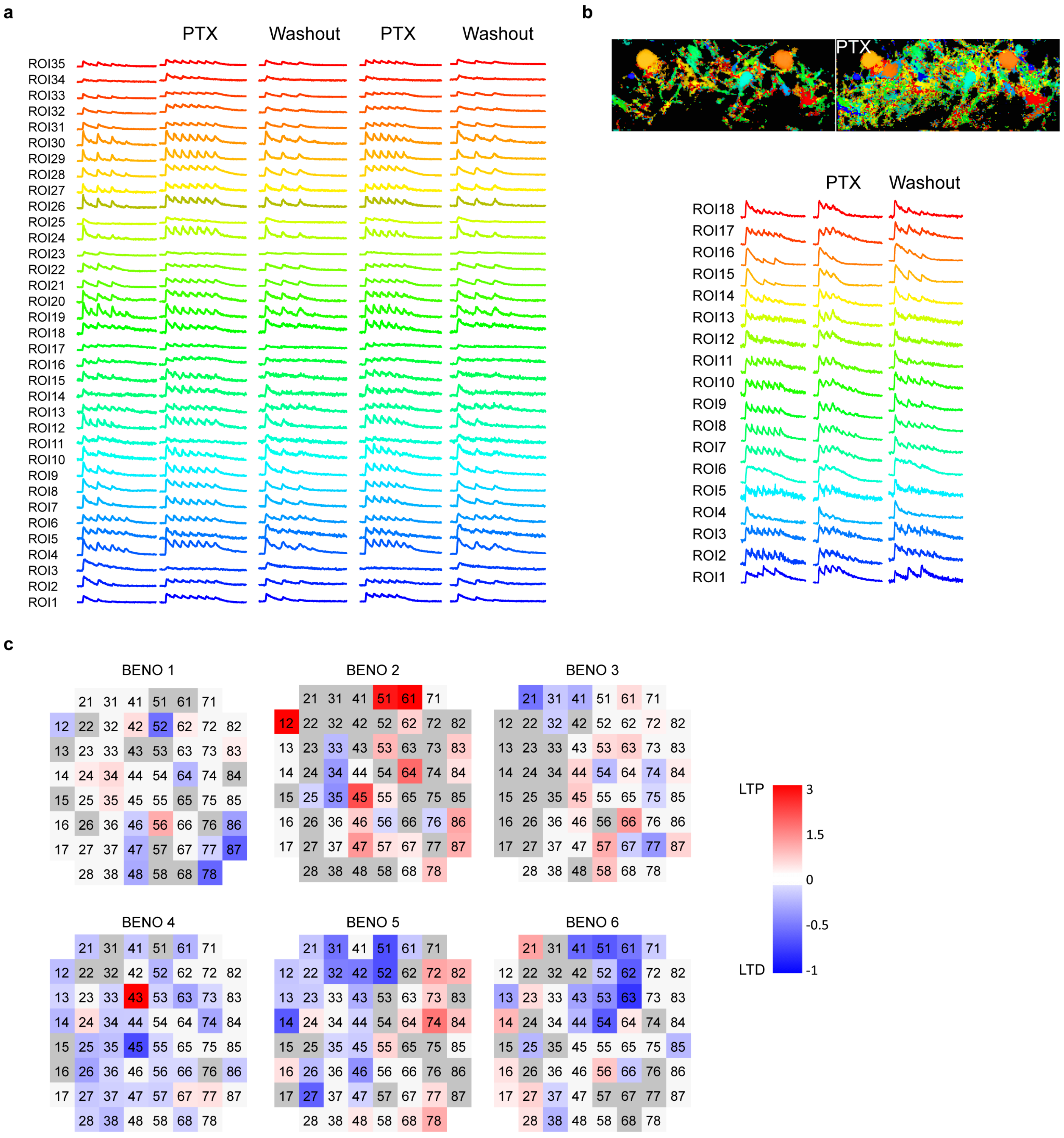

Supplementary Fig. 9| Network plasticity in BENOS. a, Calcium traces of 35 ROIs showing PPD. Upon PTX PPD is alleviated and upon washout PPD re-appeared. **b**, Calcium traces of 18 ROIs in a another BENO on d49. The observed PPD was alleviated upon PTX and upon washout re-appeared. **c**, Activity heat maps from 6 measurements for LTP induction in 6 different BENOs. Grey fields depict electrodes with no signal, white stable signal, red >15% LTP and blue >15% LTD 1h after HFS. Traces in main Figure 6f are from BENO 6.

Supplementary Tables

Supplementary Table **1**.Detailed antibody list containing respective dilutions for WmIF

Antibody	Order nr	Company	Clonality	Raised in	Dilution
Anti-GFAP	840001	Biolegend GmbH	poly	rabbit	1:500
Anti-PAX6	901301	Biolegend GmbH	poly	rabbit	1:500
Anti-Vglut1	821301	Biolegend GmbH	mono	Mouse IgG1	1:50
Anti-SYP	837101	Biolegend GmbH	mono	Mouse IgM	1:1000
Anti-Gaba(B)R2	820501	Biolegend GmbH	mono	Mouse IgG1	1:50
Anti-TUJ1	801202	Biolegend GmbH	mono	Mouse IgG2a	1:5000
Anti-MAP2	801801	Biolegend GmbH	mono	Mouse IgG1	1:4000
Anti-MAP2	188002	Synaptic systems	poly	rabbit	1:400
Anti-GluR1	PA1-46151	Thermoscientific	poly	rabbit	1:100
Anti-PSD95	810301	Biolegend GmbH	mono	Mouse IgG1	1:100
Anti-NF (H)	822601	Biolegend GmbH	poly	Chicken IgY	1:20000
Anti-GABA	A2052	Sigma	poly	rabbit	1:300
Anti-Ki67	M7240	DAKO	mono	Mouse IgG1	01:20
Anti-TH	AB152	merck millipore	poly	rabbit	1:500
Anti-Tbr2	14-4877-82	ebioscience	mono	Mouse IgG1	1:200
Anti-CTIP2	ab18465	abcam	mono	Rat IgG	1:600
Anti-MBP	836504	Biolegend GmbH	mono	Mouse IgG1	1:100
Anti-S100	287004	Synaptic systems	poly	guiney pig	1:500
Anti-Olig2	ab9610	merck millipore	poly	rabbit	1:100
Anti-CNPase	C5992	Sigma	mono	Mouse IgG1	1:100
Alexa488 anti-Goat	A-11055	Thermoscientific		Donkey	1:400
Alexa568 anti-Goat	A-11057	Thermoscientific		Donkey	1:400
Alexa647 anti-Goat	A-21447	Thermoscientific		Donkey	1:400
goat anti-chicken IgY-488	A-11039	Thermoscientific	-	goat	1:400

Supplementary Table **2**. Detailed primer sequence list

Transcript	Species	GenBank	Primer	Sequence (5' --> 3')	Fragment length (bp)	Annealing temperature
GABBR2	human	NM_005458	F	ACC AAC TTC TTC GGG GTC AC	96	60°C
			R	CAC CTC CCT GCT GTC TTG AA		
GAPDH	human	NM_002046	F	AAG GCT GTG GGC AAG GTC ATC	248	60°C
			R	GCG TCA AAG GTG GAG GAG TGG		
GFAP	human	NM_001131019	F	GCA GAT TCG AGG GGG CAA AA	104	60°C
			R	TCT GGT GAG CCTGTA TTG GT		
GRIN1	human	NM_007327	F	CGT GAG TCC AAG GCA GAG AA	80	60°C
			R	TCT TTC GCC TCC ATC AGC AG		
GPR22	human	NM_005295.2	F	ATGTTTATGACCCTCTTCCCCC	121	60°C
			R	TGCTATTGGCCCTCTGCTAAA		
MAP2	human	NM_002374.3	F	GAGAATGGGATCAACGGAGA	100	60°C
			R	CTGCTACAGCCTCAGCAGTG		
OCT4	human	NM_002701	F	CAGTGCCCGAAACCCACAC	161	60°C
			R	GGAGACCCAGCAGCCTCAAA		
PAX6	human	NM_001258465.1	F	CCCCACATATGCAGACACAC	112	60°C
			R	TCACTTCCGGGAACTTGAAC		
TBP	human	NM_003194.4	F	GCACAGGAGCCAAGAGTGAA	176	60°C
			R	TTGTTGGTGGGTGAGCACAA		
