# Supplementary material for: Developmental GABA polarity switch and neuronal plasticity in Bioengineered Neuronal Organoids
Source: Nat Commun. 2020 Jul 29;11:3791. doi: 10.1038/s41467-020-17521-w (PMC7391775; doi:10.1038/s41467-020-17521-w)
Supplement: Supplementary file 2 — Description of Additional Supplementary Files [file 41467_2020_17521_MOESM2_ESM.pdf]

Description of Additional Supplementary Files

File Name: Supplementary Data 1
Description: Gene ontology analysis of transcripts regulated in BENOs d15 vs d40.

File Name: Supplementary Movie 1
Description: Time-lapse video of overview calcium activity of BENO 1 on d21 showing partial GDP-like events.

File Name: Supplementary Movie 2
Description: Repeat time-lapse video of calcium activity in BENO 1 (the same BENO of supplementary video 1) on d27 indicating extended GDP-like events.

File Name: Supplementary Movie 3
Description: Time-lapse video of overview of calcium activity of BENO 2 on d36 showing GDP-like events.
