# Supplementary material for: Developmental GABA polarity switch and neuronal plasticity in Bioengineered Neuronal Organoids
Source: Nat Commun. 2020 Jul 29;11:3791. doi: 10.1038/s41467-020-17521-w (PMC7391775; doi:10.1038/s41467-020-17521-w)
Supplement: Supplementary file 7 — Reporting Summary [file 41467_2020_17521_MOESM7_ESM.pdf]

nature research

Corresponding author(s): Maria-Patapia Zafeiriou & Wolfram-Hubertus Zimmermann

Last updated by author(s): Jun 22, 2020

Reporting Summary

Nature Research wishes to improve the reproducibility of the work that we publish. This form provides structure for consistency and transparency in reporting. For further information on Nature Research policies, see Authors & Referees and the Editorial Policy Checklist.

Statistics

For all statistical analyses, confirm that the following items are present in the figure legend, table legend, main text, or Methods section.

n/a	Confirmed	
☐	☒	The exact sample size (*n*) for each experimental group/condition, given as a discrete number and unit of measurement
☐	☒	A statement on whether measurements were taken from distinct samples or whether the same sample was measured repeatedly
☐	☒	The statistical test(s) used AND whether they are one- or two-sided<br>*Only common tests should be described solely by name; describe more complex techniques in the Methods section.*
☒	☐	A description of all covariates tested
☐	☒	A description of any assumptions or corrections, such as tests of normality and adjustment for multiple comparisons
☐	☒	A full description of the statistical parameters including central tendency (e.g. means) or other basic estimates (e.g. regression coefficient) AND variation (e.g. standard deviation) or associated estimates of uncertainty (e.g. confidence intervals)
☐	☒	For null hypothesis testing, the test statistic (e.g. *F*, *t*, *r*) with confidence intervals, effect sizes, degrees of freedom and *P* value noted<br>*Give P values as exact values whenever suitable.*
☒	☐	For Bayesian analysis, information on the choice of priors and Markov chain Monte Carlo settings
☒	☐	For hierarchical and complex designs, identification of the appropriate level for tests and full reporting of outcomes
☒	☐	Estimates of effect sizes (e.g. Cohen's *d*, Pearson's *r*), indicating how they were calculated

Our web collection on statistics for biologists contains articles on many of the points above.

Software and code

Policy information about availability of computer code

Data collection	Confocal imaging of fixed BENOs was performed by a Zeiss LSM 710 confocal microscope equipped with ZEN 2010 software (Zen 2.3 (black edition), version 13.0.0.518). Calcium imaging was performed by standard confocal microscopy, Zeiss LSM 780. For Patch clamping an EPC-9 patch-clamp amplifier equipped with Patchmaster software (version 2x65, HEKA Electronics, Germany) was used. The data were sampled at 20 kHz and filtered at 10 kHz (four-pole Bessel) and 5.9 kHz (three-pole Bessel). MEA data for network analysis were collected by the stand alone tool AxIS Navigator (version 2.0.4.21) from Axion Biosystems using the manufacturer's Spontaneous Neural Configuration. MEA data for LTP analysis were collected by MC_Rack v3.2.1.0 software from Multi Channel Systems. qPCR data were collected with a 7900 HT Fast Real-Time PCR system by SDSv2.4 software from Applied Biosystems.
Data analysis	LSM files were read and analysed in Matlab (version 2014, version 2017, and version 2018) with scripts adopted from Matlab LSM File Toolbox (https://www.mathworks.com/matlabcentral/fileexchange/8412-lsm-file-toolbox?s_tid=FX_rc1_behav). MEA network analysis was performed by the Neural Metric Tool (version 2.5.1) and AxIS Metric Plotting Tool (version 2.2.5) from Axion Biosystems. LTP data were analysed and exported by MC_Rack v3.2.1.0. qPCR data were analysed in SDSv2.4 software from Applied Biosystems. For statistical analyses and graphical display of the data Graph Pad Prism(version 7.00 for Windows, GraphPad Software, La Jolla California USA) was used. Fastq files were mapped using TopHat (version 2.1.1) and fragments per kilobase of transcript per million (FPKM) calculated using Cufflinks (version 2.2.1). Gene Ontology (GO) analysis was performed by ClueGo plugin (version 2.5.1) in cytoscape (version 3.6.1). The visualization of data was done in R (version 3.0).

For manuscripts utilizing custom algorithms or software that are central to the research but not yet described in published literature, software must be made available to editors/reviewers. We strongly encourage code deposition in a community repository (e.g. GitHub). See the Nature Research guidelines for submitting code & software for further information.

Data

Policy information about availability of data

All manuscripts must include a data availability statement. This statement should provide the following information, where applicable:

- Accession codes, unique identifiers, or web links for publicly available datasets
- A list of figures that have associated raw data
- A description of any restrictions on data availability

The gene array datasets generated in this work have been deposited in Gene Expression Omnibus under the accession number GSE139101 (https://www.ncbi.nlm.nih.gov/geo/query/acc.cgi?acc=GSE139101). The data that support the findings of this study are available from the corresponding author upon reasonable request.

Field-specific reporting

Please select the one below that is the best fit for your research. If you are not sure, read the appropriate sections before making your selection.

☒ Life sciences ☐ Behavioural & social sciences ☐ Ecological, evolutionary & environmental sciences

For a reference copy of the document with all sections, see nature.com/documents/nr-reporting-summary-flat.pdf

Life sciences study design

All studies must disclose on these points even when the disclosure is negative.

Sample size	Sample size was empirically determined by transcriptome expression. The inter-organoid variability in terms of expression of neuronal and glial markers indicated a minimum of 3 organoids generated in two to 3 independent differentiation to be used per condition.
Data exclusions	Data were not excluded. Variability in number of biological replicates was a result of technical reasons (ie. RNA concentration too low, sample used for different analysis).
Replication	All data presented were tested in a minimum of two independent differentiation experiments to verify the reproducibility of the findings. The developmental windows of expression data, as well the staged network function were similar in the two different iPSC lines as well as in between independent differentiation runs of the same line. All replication attempts were successful with 93% of the organoids presenting similar spontaneous activity.
Randomization	Allocation of organoids was random. Organoids from different (if possible) or the same well for each condition were submitted to analysis in each differentiation run.
Blinding	There was no blinding in this study since we did not attempt to compare patient to control iPS lines.

Reporting for specific materials, systems and methods

We require information from authors about some types of materials, experimental systems and methods used in many studies. Here, indicate whether each material, system or method listed is relevant to your study. If you are not sure if a list item applies to your research, read the appropriate section before selecting a response.

Materials & experimental systems

n/a	Involved in the study
☐	☒ Antibodies
☐	☒ Eukaryotic cell lines
☒	☐ Palaeontology
☒	☐ Animals and other organisms
☒	☐ Human research participants
☒	☐ Clinical data

Methods

n/a	Involved in the study
☒	☐ ChIP-seq
☒	☐ Flow cytometry
☒	☐ MRI-based neuroimaging

Antibodies

Antibodies used

Rabbit Anti-GFAP (Biolegend GmbH , 840001), 1:500
Rabbit Anti-PAX6 (Biolegend GmbH , 901301), 1:500
Mouse Anti-Vglut1 (Biolegend GmbH , 821301), 1:50
Mouse Anti-SYP (Biolegend GmbH , 837101), 1:1000
Mouse Anti-Gaba(B)R2 (Biolegend GmbH , 820501), 1:50
Mouse Anti-TUJ1 (Biolegend GmbH , 801202), 1:5000
Mouse Anti-MAP2 (Biolegend GmbH , 801801), 1:4000

Rabbit Anti-MAP2 (Synaptic systems , 188002), 1:400
Rabbit Anti-GluR1 (Thermoscientific , PA1-46151), 1:100
Mouse Anti-PSD95 (Biolegend GmbH , 810301), 1:100
Chicken Anti-NF (H) (Biolegend GmbH , 822601), 1:20000
Rabbit Anti-GABA (Sigma , A2052), 1:300
Mouse Anti-Ki67 (DAKO , M7240), 1:200
Rabbit Anti-TH (merck millipore , AB152), 1:500
Mouse Anti-Tbr2 (ebioscience , 14-4877-82), 1:200
Rat Anti-CTIP2 (abcam , ab18465), 1:600
Mouse Anti-MBP (Biolegend GmbH , 836504), 1:100
guiney pig Anti-S100 (Synaptic systems , 287004), 1:500
Rabbit Anti-Olig2 (merck millipore , ab9610), 1:100
Mouse Anti-CNPase (Sigma , C5922), 1:100
Donkey Alexa488 anti-Goat (Thermoscientific , A-11055), 1:400
Donkey Alexa568 anti-Goat (Thermoscientific , A-11057), 1:400
Donkey Alexa647 anti-Goat (Thermoscientific , A-21447), 1:400
Goat Alexa488 anti-chicken (Thermoscientific , A-11039), 1:400

Validation

Most of the antibodies used in this study were tested and validated in a number of studies. Citations validating the antibodies are listed below:

GFAP Miyamoto et al. 2016 Nat Commun. 7: 13478;
PAX-6 Bando et al. 2016 Cereb Cortex. 26: 106; Pataskar et al. 2016 EMBO J. 35: 24; Martínez-Cerdeño et al. 2016 Cereb Cortex. 26: 374
SYP Wolf et al. 2012. J Alzheimers Dis. 32: 217; McDonnel et al 2008. Mol Cancer Ther. 7: 659
TUJ1 Flores-Otero et al. 2007 J Neurosci. 27: 14023; Barry et al. 2012. J Neurosci. 32: 6209; Jongbloets et al. 2017. Nat Commun. : 8: 14666.
Ms MAP-2 Gensel et al. 2009. J Neurosci. 29: 3956; Wang et al. 2015. PLoS One. 10: 0145441; Shelton et al. 2015. Biol. Psychiatry. 78(6):374.
Rb MAP-2 Wang et al 2016 Neuron 914: 777; Liu et al 2014 The Journal of neuroscience 3437: 12289
NF Greaves et al. 2015. Am J Pathol. 185: 2286; Zappulo et al. 2017. Nat Commun. 10.1038/s41467-017-00690-6; Ciolli Mattioli et al. 2019. Nucleic Acids Res. 47: 2560; Zhang et al. 2019. Adv Sci (Weinh). 6:1800808.
GABA Maria C Marchetto et al. 2016 Molecular psychiatry, 22: 6; Sara B Glickstein et. al 2007 Development 134: 22; Menegola et al. 2008 Neuroscience, 154: 3
Ki67 Jiang et al. 2019 In Cell Communication and Signaling, 17: 109; Radonjić et al. 2014 Front. Neuroanat. 8: 82.
TH Gyllborg et al. 2018 Stem Cell Reports 11:651; Dhandapani et al. 2018 Nature Communications 9: 1640; Lai et al. 2018
Nature Communications 9: 1066
TBR2 Rolot et al. 2018 Nature Communications 9: 4516; Silva et al. 2018 Cell 172: 1063; Wang et al. 2018 JCI 128(8): 3319;
CTIP2 Rhee et al. 2019 Cell Reports 27: 2212; Yoon et al. 2019 Nature Methods 16(1): 75; Li et al. 2017 Neuron 96: 387;
MBP Kiryu-Seo et al. 2010. J. Neurosci. 30: 6658; Gensel et al. 2009. J. Neurosci. 29: 3956; Wang et al. 2008. J. Cell. Biol. 182: 1171;
S100beta Filice et al. 2017 The Journal of comparative neurology 52515: 3266; Kobayashi et al. 2019 Cell reports 284: 979; Zhou et al., 2019 PLoS Biology 17(8):e3000086; Tertil et al., 2018 Translational Psychiatry 8: 255

OLIG2 Sorrells et al. 2019 Nature Communications 10: 27; Xiao et al. 2018 Nature Communications 9: 2865; Belle et al. 2017 Cell 169: 161
CNPase Xiao et al. 2018 Nature Communications 9: 2865; Berret et al. 2017 Nature Communications 8: 557; Berghoff et al. 2017 Nature Communications 8: 14241
Vglut1 Ku, T., Swaney, J., Park, J. et al. 2016 Nat Biotechnol 34, 973–981
GluR1 X. Huang et al. 2019; IEEE Transactions on Ultrasonics, Ferroelectrics, and Frequency Control, vol. 66, no. 5, pp. 930-938
PSD95 Brown et al. 2018 Mol Autism. 9:48
Tbr2 Thomsen et al. 2016 Nat Methods 13(1):87-93.; Anderson et al 2018 Front Immunol 9:301.

Eukaryotic cell lines

Policy information about cell lines

Cell line source(s)

The use of human induced pluripotent stem cells was according to institutional regulations (IRB approval: 10/9/15). Informed consent for the research use of the G1 (Tiburcy et al. 2017) and TC1133 iPSC lines (Baghbaderani et al. 2015) was obtained previously. The iPSC-G1 line and the TC1133 iPSC lines can be obtained from the Institute of Pharmacology and Toxicology, University Medical Center and Lonza, respectively, upon reasonable request.

Baghbaderani, B. A. et al. cGMP-Manufactured Human Induced Pluripotent Stem Cells Are Available for Pre-clinical and Clinical Applications. Stem Cell Reports 5, 647-659, doi:10.1016/j.stemcr.2015.08.015 (2015).

Tiburcy, M. et al. Defined Engineered Human Myocardium With Advanced Maturation for Applications in Heart Failure Modeling and Repair. Circulation 135, 1832-1847, doi:10.1161/CIRCULATIONAHA.116.024145 (2017).

Authentication	Both cell lines were confirmed to be karyotypically normal and are widely used in our laboratory. Regular quality control was performed by flow cytometry using TRA-1-60 (BD Biosciences, Cat 560173), OCT4 (BD Biosciences, Cat 560329) and NANOG (Miltenyi Biotec, 130-105-050) to test for pluripotency. Cells with pluripotency higher or equal to 90% were used for experiments.
Mycoplasma contamination	The cell lines were tested for mycoplasma by Mycoalert Plus (Lonza, # LT07-705) in a monthly basis. No mycoplasma contamination was detected.
Commonly misidentified lines (See ICLAC register)	No commonly misidentified cell lines were used in the study.
